# Non-native plant integration into plant-insect pollinator networks in urban parks

**DOI:** 10.1371/journal.pone.0353207

**Published:** 2026-07-14

**Authors:** Laura Matas-Granados, Alejandro Trillo, Montserrat Vilà

**Affiliations:** 1 Department of Conservation Biology and Global Change, Doñana Biological Station (EBD-CSIC), Seville, Spain; 2 Department of Plant Biology and Ecology, University of Seville, Seville, Spain; University of Murcia: Universidad de Murcia, SPAIN

## Abstract

Pollination sustains biodiversity and ecosystem functioning, yet it is increasingly threatened by biological invasions. Non-native plants frequently integrate into plant-pollinator networks, generating both positive and negative effects on native plants. However, most current knowledge derives from static or short-term studies of communities invaded by a few species, offering limited understanding of plant-pollinator dynamics in systems where many non-native species coexist under favorable environmental conditions. Here, we examined plant-insect pollinator networks in urban green areas of a Mediterranean city as a model system to evaluate the role of non-native plants in structuring plant-pollinator networks over the course of one year. We compared native and non-native plants in terms of (i) species-level parameters (normalized degree, strength, specialization, contribution to nestedness, and module roles), (ii) modularity and co-flowering patterns, (iii) persistence of interactions, and (iv) beta-diversity metrics. Non-native plants showed greater specialization and among-module connectivity, whereas native species exhibited greater species strength and within-module connectivity. Native–non-native pairs within modules co-flowered more often, suggesting simultaneous interactions that may foster both competition and facilitation. In contrast, non-native–non-native pairs displayed asynchronous flowering, potentially sustaining shared pollinators over longer periods and mitigating competition. Despite these differences, both groups contributed similarly to temporal network connectivity, and interaction turnover was unaffected by the proportion of non-native species. Overall, these findings reveal that while native and non-native plants play distinct roles in structuring plant-pollinator networks, they do not differ in their contribution to maintaining temporal connectivity. Our study underscores the importance of incorporating temporal and multispecies approaches when assessing the ecological consequences of biological invasions.

## Introduction

Pollination is a fundamental ecological process underpinning the reproduction of most flowering plants, and thus it plays a key role maintaining terrestrial ecosystem diversity and functioning [1,2]. Animal-mediated pollination supports the reproductive success of over 87.5% of angiosperm species [3], influencing both individual plant fitness and community dynamics. Given its central role in pollination, identifying the major threats to pollination and understanding their ecological consequences are crucial for biodiversity conservation.

Biological invasions, resulting from human-mediated introductions of non-native plants and pollinators, can lead to resource competition with native species and restructure pollination interactions [4,5]. Non-native species often become well-integrated into plant-pollinator communities [6–9]. Many non-native invasive plants exhibit traits such as prolonged flowering periods [10] and high floral density [11], which enhance their attractiveness to pollinators [12]. As a result, pollinators may shift their foraging preferences toward non-native plants, reducing visitation rates and pollen transfer to native species [13]. This “encroaching effect” can lead to a decline in native plant reproduction through pollen limitation, heterospecific pollen deposition, and reductions in pollen quality [14]. Conversely, non-native plants may have facilitative effects on native plants, by sustaining pollinator populations during periods of native floral scarcity, increasing overall floral abundance and diversity, or attracting more pollinators to the community [6,15,16].

Plant-pollinator networks are a powerful tool for analyzing the integration of non-native plant species into communities and assessing their ecological consequences (e.g., [17,18]). Bipartite networks provide a framework for characterizing interactions at both the species and the community level, providing insights into the ecological roles and interaction dynamics of individual plants and pollinators [19]. The presence of non-native plant species can alter network parameters at multiple scales. At the species-level, non-native plant taxa often exhibit high degree (number of interaction partners) and species strength (the sum of interaction frequencies), reflecting high attractiveness and frequent visitation by a diverse set of pollinators [4,8]. At the network-level, nestedness—where specialist species interact with subsets of the partners of generalist species [20]—may increase as non-native plants integrate into the core of the network [18]. Although, this can lead to stability in the short term, it may mask long-term disruptions to native species interactions. Conversely, modularity, which measures the degree to which the network is divided into subgroups of tightly interacting species [21], may decrease if non-native plants increase overall network connectivity and blur ecological boundaries between modules [22]. Such structural changes can ultimately reduce community resilience to disturbances and increase the risk of coextinctions [15].

Most knowledge of the effects of non-native species on plant-pollinator networks has been generated by compiling data from single points in time or aggregating interactions across entire flowering seasons [6,23,24]. Such static approaches may overlook key aspects of how mutualistic interactions fluctuate over time [25,26] and how species connect sequential networks [27]. Focusing on temporal dynamics in plant-pollinator networks allows for exploring interaction turnover—the variation in plant-pollinator interactions over time—and its components: species turnover (gain or loss of species) and interaction rewiring (changes in interactions among a stable species pool) [28–30]. Therefore, incorporating beta-diversity indexes into network analyses can clarify how non-native species reshape mutualistic interactions over time and drive the temporal assembly of interactions.

Research on non-native plant integration within plant-pollinator networks has largely focused on communities invaded by a few, highly abundant non-native species [6,13,15,24,31,32]. However, much less attention has been paid to systems that host numerous non-native plant species [33]. This distinction is important because the combined effects of many coexisting non-native species may differ from those in systems dominated by few invaders [34]. Furthermore, most studies have examined on natural or semi-natural communities, where environmental constraints—such as water scarcity or extreme temperatures— can differently affect the performance of plant species [35,36], and indirectly influence plant-pollination interactions [37]. Therefore, to better understand non-native plant integration, it is essential to examine systems with high non-native diversity and minimal environmental limitations. Urban green spaces provide such an opportunity, offering a suitable experimental setting for investigating plant-pollinator networks dominated by ornamental non-native species, the main pathway for invasive plant introductions into semi-natural peri-urban areas [38,39].

In this study, we examined urban green spaces in a Mediterranean city to understand differences between native and non-native woody plant species in structuring networks with diurnal insect pollinators throughout an entire year. We focus on Hymenoptera, Diptera, Lepidoptera and Coleoptera, which are the main pollinators visiting flowers in Mediterranean urban green areas [40]. Our specific questions and hypotheses are as follows:

1. Do non-native plant species play a different role than native species in structuring plant-pollinator networks? We hypothesize that non-native plant species will exhibit a higher number of interactions, greater connection strength, greater contribution to nestedness, and a higher capacity to connect different modules within plant-pollinator networks, due to their generalist roles in communities [5,6]. In contrast, we expect that native plant species will display a greater degree of specialization and ability to enhance connectivity within modules, as they attract more specialist pollinators than non-native plants [17,41].
2. What role do non-native vs. native plant species play in forming and maintaining modules them throughout the year? We expect that, non-native and native plant species forming modules together will not overlap temporally because of alternative flowering phenologies [42]. Therefore, the modules that both groups form with pollinators are expected to persist throughout the year.
3. Do plant-pollinator interactions involving non-native plant species connect temporal plant-pollinator networks throughout the year? We anticipate that non-native plant species, which typically flower for longer periods [8,43], will maintain the structure among temporal networks more than native species.
4. How do non-native plant species maintain interaction turnover throughout the year? We expect that a greater proportion of non-native plant species will contribute to a higher interaction turnover throughout the year, supported by their longer temporal flowering persistence [8,43].

## Methods

### Study site and sampling design

The study was conducted between October 2021 and September 2022 in 15 urban green areas (i.e., parks) within the city of Seville, in southern Spain. In this city, owing to gardening and consistently warm temperatures throughout the year, floral resources are available nearly year-round. In each park, we selected a representative 20 m-wide plot with a length that covered the entire extent of each park. In each plot, flowering ornamental woody plant species (both native and non-native) were sampled once a month. The plant and insect pollinator sampling protocol are described in [44]. In sum, in each park flowering plant species which attracted pollinators were sampled across the entire area of the park. Each month, for each plant species, pollinators were sampled in two censuses located in two randomly selected plants along the park. In a census, a 10-minute pollinator observation over a 1 m^2^ area of the flowering plant species was conducted. Within a census any Hymenoptera, Diptera (>3 mm size), Lepidoptera and Coleoptera (>3 mm size) that contacted any reproductive part of a flower was identified and counted. We avoided double counting the same individual within the 1 m^2^ area sampling area. However, we acknowledge that pseudoreplication could not be entirely excluded if, for instance, the individual leaves the sampling area and comes back. Most specimens were identified visually in the field to the lowest possible taxonomic level, except 7.5% of them that were captured for identification in the laboratory with the help of an expert (Francisco P. Molina). Total sampling was 258 hours across 64 days (S1 Table). For further detailed information regarding the sampling design, please refer to [44] (transcribed verbatim in S1 Appendix).

### Construction of plant-pollinator networks

Overall, the plant-pollinator analyses were based on 62 plant taxa (44 non-native and 16 native) (S2 Table). Although our aim was to focus at the plant species resolution level, we grouped 17 plant taxa by genus because there was a mix of species and hybrids with cryptic floral traits, which we assumed were equally attractive to pollinators [44]. These plants were visited by 8422 pollinators belonging to 173 pollinator taxa (99 Hymenoptera, 57 Diptera, 12 Lepidoptera, 5 Coleoptera), 71% identified at the species level (S3 Table).

In some parks and months, no plant-pollinator interactions were recorded or only a single plant taxon was involved, precluding network construction. To address this limitation, we grouped sampling months by plant flowering phenology, using the. Jaccard similarity index (‘vegdist’ function, *vegan* R package; [45]) to quantify compositional dissimilarities in flowering taxa across months. This analysis identified four distinct temporal clusters of three consecutive months: [1] February–April, [2] May–July, [3] August–October, and [4] November–January (S1 Fig). We used these periods in subsequent analyses to construct four temporal networks per park, except for Álvaro Diamantino (periods 1 and 4) and Miguel Mañara parks (periods 2–4), where plant diversity was insufficient (< 2 taxa).

To estimate sampling completeness of interaction diversity for each park and period, we used the Chao1 estimator of species richness [46,47]. We calculated sampling completeness as the ratio of the observed and to estimate richness of pollinators, using the *iNext* R package [48].

### The role of native and non-native plant species in network structure

To compare the roles of native and non-native plant taxa in structuring plant-pollinator networks, for each park we calculated:

(i) normalized degree—the proportion of observed interactions for each species, weighted by the total number of interactions in the network [49,50]. It reflects the diversity of interaction of a species.
(ii) species strength—the sum of dependencies a plant species has with all pollinators it interacts, providing a measure of its overall importance within the network [51].
(iii) contribution to nestedness—the extent to which individual plant species’ interactions influence the overall nested structure of the network [20,52]. Nestedness is a structural network property where specialist species tend to interact with subsets of the partners of generalist species.
(iv) specialization (d’)—strength to which a plant species interacts with a limited number of pollinator species. High levels of specialization for a plant species occur when it relies on a specific pollinator species, while low levels occur when it interacts with a wide range of pollinators [53].
(v) modularity—the tendency of the network to be compartmentalized into distinct units, where species interact more frequently within their own module than with those in other modules [21,54]. In relation to modularity, we examined two species-level parameters:
  a. within-module degree *z—*measurement of how well-connected a species is to other species within its own module [21]
  b. among-module connectivity *c*—measurement of how a species in a module is linked to other modules [21]

Except for modularity, we evaluated the influence of native vs. non-native plant taxa on these parameters using generalized linear mixed models (GLMMs) with Gaussian error distributions, fitted with the *lme4* R package [55]. Each parameter was treated as a dependent variable. Predictor variables included: (a) plant origin (native vs. non-native), (b) period, (c) floral availability (number of flowers per m^2^ per plant taxon), and (d) network size (number of plant and pollinator taxa). We included park as a random factor to account for species composition differences among parks. Parks also have different sizes (mean = 0.17 km^2^, range = 0.003–0.511 km^2^), and are located at varying distances from the city edge to the center (mean = 1.5 km, range = 0.0–3.4 km). We log-transformed normalized degree and species strength prior to analyses to meet model assumptions.

We calculated modularity for each park with the ‘computeModules’ function using the Beckett algorithm [56]. As some temporal networks were small and low modularity is expected in small networks [21], we aggregated all temporal networks per park to calculate modularity. To assess the statistical significance of modularity for each plant-pollinator network, we used a null model analysis, where we compared the observed modularity against the expectation of 1000 randomly constructed networks using the r2dtable algorithm of the ‘nullmodel’ function in the *bipartite* R package [57]. We calculated both *z*-scores and *p*-values to test whether the observed modularity of each network significantly differed from the average modularity of 1000 random networks [57,58]. To assess whether native and non-native plant taxa differed in their contribution to modularity, we fitted GLMMs with (a) plant origin, (b) flowering duration (1–4 periods), (c) floral availability, and (d) network size as predictors. For within-module degree (*z*), we used a Gaussian error distribution while for among-module connectivity (*c*), we used a beta error distribution. Park was included as a random factor in the models.

We assessed potential collinearity among predictors for models using generalized variance inflation factors (GVIFs) [59]. All values for all models were below 1.6, indicating no problematic collinearity among predictors. For all parameters, we compared alternative models using Akaike’s information criterion (AIC) to choose the best model among all-possible combinations of predictors, with the most complex model including the interaction between all four predictors. Models were compared using ΔAIC as the difference between the AIC value of the model and the lowest AIC observed among the candidate set (ΔAIC = AICᵢ − AIC_min_). Models with ΔAIC > 2 indicated that the worst model had no support and could be omitted [60]. Models with ΔAIC ≤ 2 were considering strongly supported. When more than one model had ΔAIC ≤ 2, we decided showing the results of the model that included plant origin.

### Native and non-native plant taxa forming modules and co-flowering throughout the year

To assess whether plant taxa forming modules together overlap in their flowering phenology (hereafter co-flowering), and whether this varies with plant origin, we used the following approach:

(i) Considering the modularity of each park (see previous section), we identified plant taxa and their origin within each module.
(ii) Within each module, we generated all possible pairwise combinations of plant taxa. For example, if a module consisted of three plant taxa (e.g., plant_1_, plant_2_ and plant_3_), the resulting plant-plant pairs would be: plant_1_-plant_2_, plant_1_-plant_3_, plant_2_-plant_3_.
(iii) For each plant-plant pair, we recorded the number of periods (0–4) in which both plant taxa co-flowered and classified pair origin as native (both native taxa), non-native (both non-native), and mixed (one native, one non-native). This allowed us to quantify, for each module, the frequency of co-flowering among plant-plant pairs and their associated origin classification.
(iv) To test whether co-flowering among co-modular plant taxa differs according to pair origin, we fitted GLMMs with a Poisson error distribution. Number of co-flowering periods was the dependent variable, while pair origin was included as the predictor. We included module nested within park as a random factor.

### Temporal network connectivity considering plant origin

To examine which type of plant-pollinator interaction was predominant throughout the year and therefore played a key role in connecting temporal networks, we built a binary interaction A_*mn*_ matrix for each park, where *m* represents each temporal period (1 –4) and *n* corresponds to the plant-pollinator interactions observed during the year. Cells were coded as 1 if interaction *n* occurred in period *m*, and as 0 otherwise. Using these matrices, we calculated betweenness centrality (*BC*) for each interaction [61], to quantify its importance in connecting temporal networks. Interactions in which *BC* > 0 were considered to contribute to connecting temporal networks. To test whether differences in *BC* of interactions varied based on the origin of the associated plant taxa, we performed non-parametric pairwise comparison using the Kruskal-Wallis test [62].

### Contribution of non-native plant taxa to temporal beta-diversity

To investigate the contribution of non-native plant taxa to interaction beta-diversity (i.e., turnover of interactions) throughout the year, we estimated the turnover of plant–pollinator interactions (β_*WN*_) using binary temporal networks to calculate the Jaccard similarity index among them. Because overall interaction turnover (β_*WN*_) is driven by several ecological processes, we first partitioned it as: β_*WN*_ = β_*ST*_ + β_*OS*_, where β_*ST*_ denotes interaction turnover due to species turnover, that is changes in interaction as plant and/or pollinator taxa become present or absent over time, and β_*OS*_ denotes interaction turnover due to interaction rewiring, that is changes in interactions due to changes in interacting partners [43,63]. We calculated β_*WN*_ and its components for sequential pairwise combinations of temporal plant-pollinator interaction networks (i.e., Period 1 – Period 2, Period 2 – Period 3, Period 3 – Period 4, and Period 4 – Period 1) using the ‘betalinkr’ function in the *bipartite* R package [29]. For each pairwise comparison, we also quantified changes in the proportion of non-native plant taxa, as a measure of variation of non-native plant taxa presence over time.

To assess whether changes in β_*WN*_ and its components were associated with changes in non-native plant proportion, we fitted GLMMs with a beta error distribution. Each beta-diversity index was treated as the dependent variable against the difference in non-native plant proportion. Pairwise temporal comparisons (i.e., temporal network 1 – temporal network 2, etc.) were nested within park as a random factor, to account for potential spatial and temporal pseudoreplication.

We calculated all network parameters, betweenness centrality, and beta-diversity indexes with the inclusion of *Viburnum* spp., but we excluded this taxon in models where plant origin was included, as *Viburnum* spp. comprised both native and non-native species that were indistinguishable at a glance. Network metrics (i.e., species-level network parameters, betweenness centrality for temporal connectors and modularity) were estimated using the *bipartite* R package [64]. All analyses were performed in R version 4.5.0 [65].

## Results

### Overall characteristics of plant-pollinator networks

We recorded 8,422 plant-pollinator interactions across parks throughout the year. The mean number of interactions per park was 217.0 ± 111.0 (± SD) in period 1, 190.0 ± 116.0 in period 2, 90.5 ± 48.2 in period 3, and 73.6 ± 54.5 in period 4. Overall, we observed a total of 1,011 different plant-pollinator interactions, with an average of 27.3 ± 18.3 per park in period 1, 27.3 ± 14.4 in period 2, 8.87 ± 4.41 in period 3, and 7.17 ± 4.84 in period 4 (S1 Table; S2 Fig). Across parks and periods, non-native taxa represented 77% ± 25% of plant species and accounted for 70% of all plant-pollinator interactions. Sampling completeness for pollinator diversity averaged 65% ± 6% (S4 Table). All parks showed significant modularity. Mean modularity was 0.396 ± 0.064 (*z* = 25.74 ± 9.69, *p* = < 0.001), with on average 6 ± 2 modules and 10.49 ± 2.79 taxa (2.80 ± 1.09 plant taxa and 7.59 ± 2.11 pollinator taxa) by park (S5 Table). On average, 83.21% ± 17.76% of plant taxa in each module were non-native (S3 Fig).

### The role of native and non-native plant species in network structure

**Fig 1:**
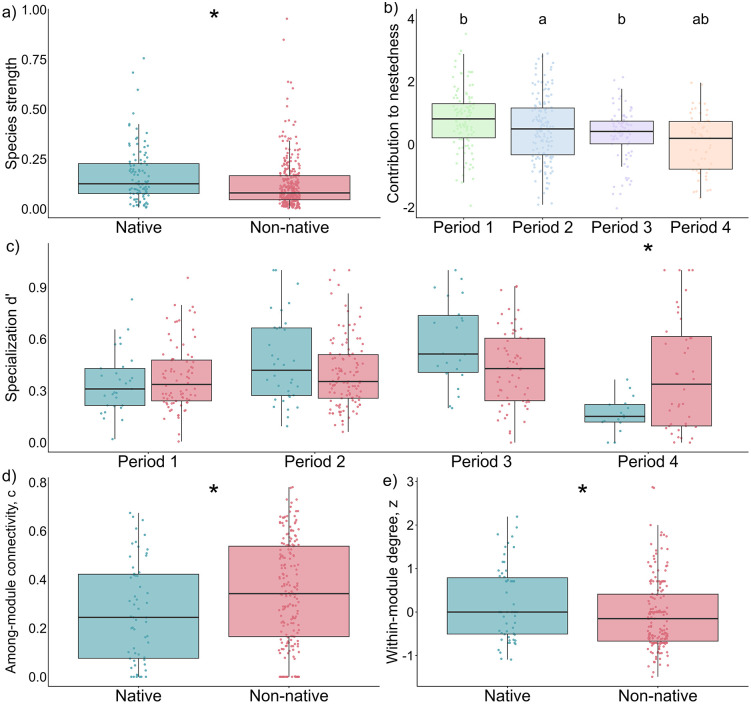
The role of native and non-native plant taxa in structuring plant-pollinator networks in Seville’s urban parks. Boxes represent the 25th to 75th percentiles, the central line is the median. Different letters in contribution to nestedness denote significant differences among periods. (*) Significant differences between native and non-native plant taxa (ANOVA; Tukey *post-hoc* multiple comparisons, *p* < 0.05).

Native and non-native plant taxa played distinct roles in structuring plant-pollinator networks throughout the year, except for normalized degree and contribution to nestedness (Fig 1). The best supported models included: (i) period, floral availability, and network size for normalized degree; (ii) plant origin, period, floral availability, and network size for species strength; (iii) period, floral availability, and network size for contribution to nestedness; (iv) plant origin, period, and their interaction for specialization; (v) plant origin and flowering length for within-module degree *z*; and (vi) plant origin, network size, and the interaction between flowering length and floral availability for among-module connectivity *c* (Table 1; S6 Table).

**Table 1:** Summary of the best model for each species-level network parameter. The level “Period 3” for the predictor “Period” and the level “Native” for the predictor “Plant origin” were used as reference level when any of these two predictors was included in the model. Statistically significant terms are highlighted in bold. “+”: marginal significant terms.

Parameter	Coefficient	Value	Standard error	t-value	p-value
Normalized degree	**Intercept**	**−0.793**	**0.096**	**−8.217**	**<0.001**
	Period 1	0.063	0.099	0.638	0.524
	Period 2	−0.055	0.106	−0.522	0.602
	Period 4	0.070	0.102	0.690	0.491
	**Scale(Floral availability)**	**0.258**	**0.030**	**8.642**	**<0.001**
	**Network_size**	**−0.025**	**0.003**	**−7.257**	**<0.001**
Species strength	**Intercept**	**−0.980**	**0.174**	**−5.631**	**<0.001**
	**Non-native**	**−0.308**	**0.107**	**−2.887**	**0.004**
	Period 1	0.164	0.155	1.056	0.291
	Period 2	0.112	0.167	0.670	0.503
	Period 4	−0.040	0.155	−0.260	0.795
	**Scale(Floral availability)**	**0.205**	**0.045**	**4.509**	**<0.001**
	**Network_size**	**−0.041**	**0.006**	**−7.052**	**<0.001**
Contribution to nestedness	Intercept	−0.194	0.169	−1.152	0.250
	Period 1	−0.042	0.164	−0.258	0.797
	**Period 2**	**−0.496**	**0.177**	**−2.796**	**0.005**
	Period 4	−0.046	0.162	−0.286	0.775
	**Scale(Floral availability)**	**0.295**	**0.047**	**6.270**	**<0.001**
	**Network_size**	**0.027**	**0.006**	**4.320**	**<0.001**
Specialization	**Intercept**	**0.574**	**0.049**	**11.616**	**<0.001**
	**Non-native**	**−0.126**	**0.055**	**−2.307**	**0.022**
	**Period 1**	**−0.252**	**0.061**	**−4.138**	**<0.001**
	**Period 2**	**−0.117**	**0.059**	**−1.982**	**0.048**
	**Period 4**	**−0.438**	**0.071**	**−6.179**	**<0.001**
	**Non-native:Period 1**	**0.203**	**0.071**	**2.85**	**0.005**
	Non-native:Period 2	0.087	0.068	1.277	0.202
	**Non-native:Period 4**	**0.405**	**0.083**	**4.858**	**<0.001**
within-module degree, *z*	**Intercept**	**−0.446**	**0.130**	**−3.430**	**<0.001**
	**Non-native**	−0.206	0.108	−1.913	+0.057
	**Flowering length**	**0.373**	**0.052**	**7.215**	**<0.001**
among-module connectivity, *c*	**Intercept**	**−3.072**	**0.424**	**−7.243**	**<0.001**
	**Non-native**	0.354	0.184	1.921	+0.055
	**Flowering length**	**0.596**	**0.097**	**6.163**	**<0.001**
	**Scale(Floral availability)**	**0.745**	**0.201**	**3.715**	**<0.001**
	**Network_size**	**0.013**	**0.005**	**2.630**	**0.009**
	**Flowering length:Scale(Floral availability)**	**−0.270**	**0.065**	**−4.155**	**<0.001**

Native plant taxa had greater species strength and higher within-module degree values than non-native taxa (Table 1; Fig 1a, e), whereas non-native plants exhibited greater specialization in period 4 and higher among-module connectivity (Fig 1c, d). Contribution to nestedness was significantly higher in periods 1, 3, and 4 than in period 2, independent of plant origin (Fig 1b). Normalized degree, species strength, and contribution to nestedness increased with flower availability, whereas normalized degree and species strength decreased, and contribution to nestedness increased, with larger network size (Table 1; S4 Fig). Finally, longer flowering duration was associated with higher within-module degree *z* and among-module connectivity *c*, except in taxa with the highest flower availability where prolonged blooming reduced *c* (S5 Fig).

### Native and non-native plant taxa forming modules and co-flowering throughout the year

**Fig 2:**
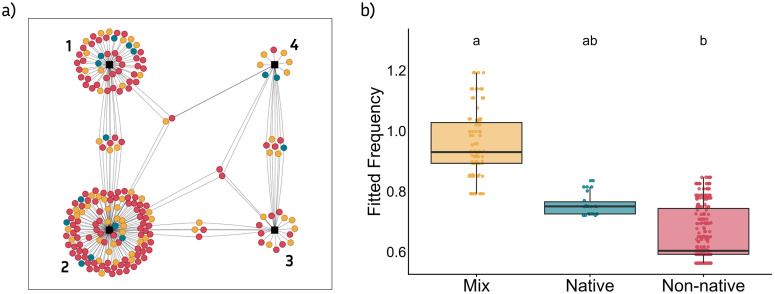
Co-flowering of plant-plant pairs forming modules in parks. a) Periods (1–4) in which plant-plant pairs co-flowered across all parks (for each park separately, see S6 Fig). Black squares denote the periods; circles represent co-flowering plant-plant pairs colored by origin (yellow: mixed, one native and one non-native; red: both non-native; and blue: both native. b) Frequency of co-flowering plant-plant pairs forming modules by origin. Different letters indicate significant differences between plant-plant pair origins (ANOVA; Tukey *post-hoc* multiple comparisons, *p* < 0.01).

In general, most plant-plant pairs forming modules co-flowered during a single period (73.17% ± 32.12 [mean ± SD]), fewer in two (10.34% ± 8.63) or three periods (3.16% ± 6.96), and none in all four periods (Fig 2a; S6 Fig). There were significant differences in the co-flowering frequency of plant taxa by pair origin. Specifically, mixed (native‒non-native) pairs within a module tended to co-flower more frequently than non-native‒non-native pairs (Fig 2b; S7 Table).

### Temporal network connectivity considering plant origin

Approximately 12.5% ± 3.65% of all plant-pollinator interactions played a role in connecting the networks through time. However, there were no significant differences in betweenness centrality (*BC*) with regard to the origin of the plant taxa involved in the interactions (*BC* interactions of native plant taxa = 0.007 ± 0.007, *BC* interactions of non-native plant taxa = 0.014 ± 0.014, Kruskal–Wallis chi-squared = 0.379, *p* = 0.538). Therefore, interactions involving non-native plant taxa had the same potential to connect temporal networks as interactions involving native plant taxa.

### Contribution of non-native plant taxa to temporal beta-diversity

Interaction composition of plant-pollinator networks varied across temporal periods (S7 Fig). Most interactions were unique to one period (87.53% ± 3.65 [mean ± SD]), followed by their presence in two (10.98% ± 2.96), three (1.29% ± 1.24), and all four periods (0.20% ± 0.44) (S7 Fig). In general, interaction turnover between consecutive temporal networks was high (β_*WN*_ = 0.92 ± 0.02). Species turnover significantly contributed more to interaction turnover across periods than interaction rewiring (β_*ST*_ = 0.80 ± 0.06; β_*OS*_ = 0.12 ± 0.05; *t*-value = 39.41, *p*-value < 0.001), with higher values between periods 1–2 (0.88 ± 0.06) and periods 4–1 (0.86 ± 0.08) than between periods 2–3 (0.76 ± 0.12) and periods 3–4 (0.75 ± 0.10). However, differences in the proportion of non-native plant taxa between periods did not explain changes in interaction turnover, species turnover, or interaction rewiring (Table 2).

**Table 2:** Summary of generalized linear mixed model (GLMM) with each β-component as the dependent variable. Statistically significant terms are highlighted in bold.

Beta component	Coefficient	Value	Standard	z-value	p-value
Interaction turnover (β_*WN*_)	**Intercept**	**2.515**	**0.093**	**27.038**	**<0.001**
	Difference percentage non-native plant taxa	−0.145	0.524	−0.277	0.782
Species turnover (β_*ST*_)	**Intercept**	**1.456**	**0.091**	**16.063**	**<0.001**
	Difference percentage non-native plant taxa	−0.372	0.528	−0.704	0.481
Interaction rewiring (β_*OS*_)	**Intercept**	**−2.226**	**0.138**	**−16.189**	**<0.001**
	Difference percentage non-native plant taxa	−0.330	0.858	−0.385	0.701

## Discussion

Our study examining year-round plant-pollination interactions in urban green areas indicate that native and non-native plants differ on how they integrate into the plant-pollinator network and in their modules. However, native and non-native plants have similar capacities as temporal network connectors, and on their contribution to interaction turnover

Non-native plant species play a different role than native species in structuring plant-pollinator networks. We expected non-native plants to show greater strength and lower specialization due to their generalist behavior. However, contrary to our hypothesis, non-native plants, such as *Pyracantha coccinea* or *Lantana* spp., displayed greater specialization than native plants during resource-scarce periods. This pattern supports previous findings that non-native species can act as key floral resource providers during periods of phenological mismatch or reduced floral abundance (e.g., 7), especially in systems dominated by several non-native species [41,43]. In contrast, native plants exhibited greater species strength (e.g., *Salvia rosmarinus*), indicating that a wide range of pollinators depend on them when available, highlighting their role in sustaining network stability [53].

As expected, non-native plants showed higher among-module connectivity (e.g., *Agapanthus africanus*), whereas natives exhibited higher within-module degree (e.g., *Salvia rosmarinus*). Elevated among-module connectivity in non-natives aligns with their generalist role in plant-pollinator networks [15]. Networks with highly interconnected modules may be more robust to random species loss but more vulnerable if key connectors—here, non-native plants—are removed [9]. In contrast, native plants tended to act as module hubs—species highly connected to pollinators within their own module [21]. The removal of native species could fragment the modules they dominate, but with limited cascading effects in adjacent modules, thus preventing network collapse.

Because many regional analyses have shown differences in flowering phenology between native and non-native plants [42], we hypothesized co-flowering native and non-native plants not to form modules. However, native–non-native pairs forming modules tended to co-flower more frequently than non-native–non-native pairs. This is illustrated by examples such as the pair *Lavandula* spp. (native) and *Lantana* spp. (non-native) which co-flowered during three of the four periods. These findings indicate that native and non-native plants interact more simultaneously with their “preferred” pollinators than do the assemblage of non-native species. Given that phenological overlap is a key factor shaping pollinator-mediated interactions among plants [16], this result suggests that non-native species may promote both competition and facilitation for pollinators. Co-occurring non-native and native species could reduce pollinator visitation to natives, ultimately decreasing reproductive success [16]. On the other hand, non-native species may provide floral resources for the pollinator community, increasing pollinator abundance and diversity, and thus having a magnet effect on co-occurring native plants [6,66]. In contrast, the asynchronous patterns observed in non-native–non-native pairs in their interactions with their preferred pollinators could indicate that they help maintain the presence of these pollinators over longer periods of the year while also potential reduce direct competition among non-native plants.

The frequent co-flowering of native–non-native pairs, alongside the relative scarcity of synchronous non-native–non-native pairs, may play an important role in structuring urban pollination networks. To clarify the balance of facilitative and competitive interactions shaping these networks it would require [67–69].

The temporal turnover of interactions across consecutive periods was very high. Such high temporal variability is a common feature of ecological networks, as interaction patterns change continuously with community composition, phenology, and environmental conditions [30,70]. Species turnover contributed more strongly to temporal changes than interaction rewiring, mirroring results from natural systems [43].

Species turnover was particularly high between the first two periods of the year, likely reflecting phenological mismatches and stronger seasonal shifts in floral and pollinator communities at this time (see Supplementary material Trillo *et al.* 2026 for comparisons of phenologies of plants and pollinators) [71]. These changes in phenology could drive the temporal segregation of plant-pollinator interactions, reducing the number of species and interactions that persist across several periods [72]. This high turnover aligns with the finding that only a few interactions connected temporal networks over the course of the year. This will be the case for some interactions between *Apis mellifera* and *Xylocopa violacea* with both native (e.g., *Lavandula* spp.) and non-native plants (e.g., *Duranta erecta*), underscoring the transient nature of most plant–pollinator interactions.

However, contrary to our expectation, changes in interaction and species turnover cannot be attributed to differences in the proportion of non-native plants between periods. This finding, together with the lack of differences in betweenness centrality, suggests that while native and non-native plants may differ in their structural contributions to certain species-level network parameters and network modularity, they do not assume distinct roles in maintaining temporal connectivity. Instead, network dynamics appear independent of variation in plant origin, likely reflecting the generalist behavior of many urban pollinators throughout the year [73].

In our urban green areas, other factors such as variation in floral traits (e.g., flower morphology, nectar availability, and reward predictability), may play a more influential role in shaping interaction dynamics than plant origin per se [74]. In particular, future studies should investigate differences in traits that mediate pollinator accessibility and attractiveness, contributing to both species turnover and interaction rewiring.

## Conclusions

Urban parks represent artificial assemblages of ornamental native and non-native plants that provide floral resources to pollinators. Our study demonstrates that non-native and native plant species contribute differently to structuring plant-pollinator networks and maintaining modules throughout the year, potentially suggesting both competitive and facilitative effects. However, both groups of plants similarly connect temporal plant-pollinator networks.

Many natural and seminatural communities are highly invaded by a few non-native plants that coexist with a wide diversity of native species. Nevertheless, increasing the intensity of global change, economic development and human movement are accelerating introduction rates and decreasing biogeographical barriers to non-native species establishment and spread [75,76]. In this context, understanding how non-native plants integrate into urban plant-pollinator networks provides a valuable model for predicting how highly invaded communities could affect natural plant-pollinator networks in the future.

## Supporting information

S1 Table Sampling effort and total number of different plants, pollinators and interactions by park. (PDF)

S2 Table List of plant taxa sampled in urban green areas of Seville. (PDF)

S3 Table List of pollinator taxa sampled in urban green areas of Seville. (PDF)

S4 Table Sampling completeness of interaction diversity in census of each park in each period and in total. (PDF)

S5 Table Modularity characteristics of each park. (PDF)

S6 Table Summary of generalized linear mixed models (GLMMs) with species-level network parameters against plant taxa origin, period, floral availability, network size, and flowering length. Best GLMM is highlighted in bold by each speces-level network parameter. When more than one model were the best, we showed in results the model which includes plant origin (for within-module degree, z).
(PDF)

S7 Table Summary of generalized linear mixed model (GLMM) with ‘number of periods in which each plant-plant pair co-flowered’ as the dependent variable. Statistically significant terms are highlighted in bold.
(PDF)

S1 Fig Dendogram showing flowering plant species phenology richness similarity (Jaccard similarity matrix) among months. Censused months where grouped according to their similarity in flowering species, obtaining four separate clusters (periods).
(PDF)

S2 Fig Representation of plant-pollinator networks over periods. Data were pooled across all parks for visual clarity. Each subpanel represents each period (Period 1: February-March; Period 2: May-July; Period 3: August-October; Period 4: November-January).
(PDF)

S3 Fig Modularity *M* of each park. Black boxes represent modules. Darker colors correspond to higher frequency of interactions. Red tones correspond to non-native plant taxa, blue tones, to native plant taxa and green tones to *Viburnum* spp. A: Alamillo, B: Álvaro Diamantino Vellisco, C: Amate, D: Los Bermejales, E: José Celestino Mutis, F: Federico García Lorca, G: Infanta Elena, H: Jardines de la Buhaira, I: Jardines del Guadalquivir, J: Jardines del Valle, K: José María de los Santos, L: Maria Luisa, M: Don Miguel Mañara, N: Parque de los Príncipes, O: Tamarguillo.
(PDF)

S4 Fig Regression model fits for normalized degree, species strength and contribution to nestedness against a) total flower availability and b) network size using *visreg* R package (Breheny & Burchett, 2017). (PDF)

S5 Fig Regression model fits for a) within-module degree z against flowering length and b) among-module connectivity c against the interaction between flowering length using visreg R package (Breheny & Burchett, 2017). (PDF)

S6 Fig Representation of co-flowering of plants that form modules together throughout the four temporal periods (from 1 to 4) for each park. Circles are plant-plant pairs that co-flowering in each period. Colours show the plant taxa origin: yellow: mix (one native and one non-native), red: non-native (both non-native) and blue native (both native). A: Alamillo, B: Álvaro Diamantino Vellisco, C: Amate, D: Los Bermejales, E: José Celestino Mutis, F: Federico García Lorca, G: Infanta Elena, H: Jardines de la Buhaira, I: Jardines del Guadalquivir, J: Jardines del Valle, K: José María de los Santos, L: Maria Luisa, M: Don Miguel Mañara, N: Parque de los Príncipes, O: Tamarguillo.
(PDF)

S7 Fig Temporal networks of plant-pollinator interactions across the parks of Seville. Black squares denote the four temporal sampling periods (1–4). Each circle represents a unique pairwise interaction between a plant taxon and a pollinator taxon in a single period, while triangles represent interactions shared among periods. Colors indicate the origin of the plant taxon involved in each interaction (red: non-native, blue: native). Grey lines connect interactions to the periods in which they were recorded. Each subpanel represents a park: A: Alamillo, B: Álvaro Diamantino Vellisco, C: Amate, D: Los Bermejales, E: José Celestino Mutis, F: Federico García Lorca, G: Infanta Elena, H: Jardines de la Buhaira, I: Jardines del Guadalquivir, J: Jardines del Valle, K: José María de los Santos, L: Maria Luisa, M: Don Miguel Mañara, N: Parque de los Príncipes, O: Tamarguillo. Network graphs were built using the visNetwork R package (Almende *et al.* 2025).
(PDF)

S1 Appendix Sampling design extracted verbatim from Trillo *et al.* (2026). (PDF)
