# Supplementary material for: Non-native plant integration into plant-insect pollinator networks in urban parks
Source: PLoS One. 2026 Jul 14;21(7):e0353207. doi: 10.1371/journal.pone.0353207 (PMC13367714; doi:10.1371/journal.pone.0353207)
Supplement: S1 Table — (PDF) [file pone.0353207.s001.pdf]

Table S1. Sampling effort, total number of plant and pollinator taxa and interactions by park.

Park	Total sampling time (min)	Time census per period (min)				Mean time census per plant species (± standard deviation)	Nº plants	Nº pollinators	Nº interactions
		1	2	3	4				
Alamillo	760	290	160	130	180	54 ± 45	14	49	98
Álvaro Diamantino Vellisco	220	40	120	60	0	37 ± 12	6	20	32
Amate	1400	470	540	210	180	50 ± 42	28	57	211
Bermejales	580	190	140	70	180	58 ± 38	10	51	102
José Celestino Mutis	1240	430	440	190	180	44 ± 38	28	40	155
Federico García Lorca	620	100	200	220	100	69 ± 50	10	37	79
Infanta Elena	610	290	160	70	90	41 ± 28	17	50	103
Jardines de la Buhaira	530	210	190	60	70	48 ± 31	11	37	83
Jardines del Guadalquivir	1060	380	400	170	110	39 ± 32	27	64	192
Jardines del Valle	890	300	270	200	120	56 ± 37	16	44	129
Jose María de los Santos	620	220	150	130	120	52 ± 50	12	45	108
María Luisa	1130	320	420	260	130	43 ± 26	26	45	177
Don Miguel Mañara	130	60	30	40	0	33 ± 15	4	13	18
Parque de los Príncipes	1320	330	560	310	120	53 ± 41	25	52	190
Tamarguillo	950	280	380	170	120	50 ± 25	19	66	154
