# Supplementary material for: Non-native plant integration into plant-insect pollinator networks in urban parks
Source: PLoS One. 2026 Jul 14;21(7):e0353207. doi: 10.1371/journal.pone.0353207 (PMC13367714; doi:10.1371/journal.pone.0353207)
Supplement: S2 Table — (PDF) [file pone.0353207.s002.pdf]

Table S2. List of plant taxa sampled in urban green areas of Seville.

Family	Species name	Plant origin	Flower colour
Fabaceae	*Acacia* spp.	Non-native	Yellow
Acanthaceae	*Acanthus mollis*	Non-native	White and purple
Amaryllidaceae	*Agapanthus africanus*	Non-native	White
Ericaceae	*Arbutus unedo*	Native	White
Fabaceae	*Bauhinia* spp.	Non-native	White and pink
Malvaceae	*Brachychiton populneus*	Non-native	Red and green
Bignoniaceae	*Catalpa bignonioides*	Non-native	White and purple
Malvaceae	*Ceiba* spp.	Non-native	White and pink
Fabaceae	*Ceratonia siliqua*	Native	Yellow
Fabaceae	*Cercis siliquastrum*	Non-native	Pink
Solanaceae	*Cestrum* spp.	Non-native	Purple
Cistaceae	*Cistus albidus*	Native	Pink
Rutaceae	*Citrus* x *aurantium*	Non-native	White
Rosaceae	*Cotoneaster* spp.	Non-native	White
Rosaceae	*Crataegus monogyna*	Native	White
Fabaceae	*Cytisus scoparius*	Native	Yellow
Hydrangeaceae	*Deutzia scabra*	Non-native	White
Malvaceae	*Dombeya* spp.	Non-native	Pink
Verbenaceae	*Duranta erecta*	Non-native	White and purple
Boraginaceae	*Heliotropium arborescens*	Non-native	White and purple
Solanaceae	*Iochroma cyaneum*	Non-native	Purple
Bignoniaceae	*Jacaranda mimosifolia*	Non-native	Purple
Acanthaceae	*Justicia adhatoda*	Non-native	White and purple
Sapindaceae	*Koelreuteria paniculata*	Non-native	Yellow
Lythraceae	*Lagerstroemia* spp.	Non-native	White and pink
Malvaceae	*Lagunaria patersonia*	Non-native	Pink
Verbenaceae	*Lantana* spp.	Non-native	Yellow and pink
Lamiaceae	*Lavandula* spp.	Native	Purple
Oleaceae	*Ligustrum* spp.	Non-native	White
Caprifoliaceae	*Lonicera japonica*	Non-native	White
Scrophulariaceae	*Myoporum laetum*	Non-native	White
Myrtaceae	*Myrtus communis*	Native	White
Berberidaceae	*Nandina domestica*	Non-native	White
Fabaceae	*Parkinsonia aculeata*	Non-native	Yellow
Lamiaceae	*Phlomis* spp.	Native	Yellow
Rosaceae	*Photinia serratifolia*	Non-native	White
Phytolaccaceae	*Phytolacca dioica*	Non-native	White
Pittosporaceae	*Pittosporum* spp.	Non-native	White
Plumbaginaceae	*Plumbago auriculata*	Non-native	Blue
Bignoniaceae	*Podranea ricasoliana*	Non-native	White and pink
Rosaceae	*Prunus cerasifera*	Non-native	White
Rosaceae	*Pyracantha coccinea*	Non-native	White
Rosaceae	*Pyrus bourgaeana*	Native	White
Fabaceae	*Retama sphaerocarpa*	Native	Yellow
Rosaceae	*Rhaphiolepis indica*	Non-native	White
Fabaceae	*Robinia pseudoacacia*	Non-native	White
Lamiaceae	*Salvia officinalis*	Non-native	White and purple
Lamiaceae	*Salvia rosmarinus*	Native	Blue
Lamiaceae	*Salvia yangii*	Non-native	Purple
Asteraceae	*Santolina chamaecyparissus*	Native	Yellow
Rosaceae	*Spiraea* spp.	Non-native	White
Fabaceae	*Styphnolobium japonicum*	Non-native	White
Tamaricaceae	*Tamarix* spp.	Native	White and pink

Family	Species name	Plant origin	Flower colour
Lamiaceae	*Teucrium fruticans*	Native	White and purple
Fabaceae	*Tipuana tipu*	Non-native	Yellow
Caprifoliaceae	*Vesalea* spp.	Non-native	White
Viburnaceae	*Viburnum* spp.	Non-native	White
Apocynaceae	*Vinca major*	Native	White and purple
Lamiaceae	*Vitex agnus-castus*	Native	White and purple
Fabaceae	*Wisteria sinensis*	Non-native	White and purple
Asparagaceae	*Yucca* spp.	Non-native	White
