# Supplementary material for: Non-native plant integration into plant-insect pollinator networks in urban parks
Source: PLoS One. 2026 Jul 14;21(7):e0353207. doi: 10.1371/journal.pone.0353207 (PMC13367714; doi:10.1371/journal.pone.0353207)
Supplement: S3 Table — (PDF) [file pone.0353207.s003.pdf]

Table S3. List of pollinator taxa sampled in urban green areas of Seville.

Order	Family	Species name
Hymenoptera	Apidae	*Amegilla albigena*
Hymenoptera	Apidae	*Amegilla fasciata*
Hymenoptera	Apidae	*Amegilla quadrifasciata*
Diptera	Bombyliidae	*Amictus* sp1
Hymenoptera	Andrenidae	*Andrena agilissima*
Hymenoptera	Andrenidae	*Andrena bimaculata*
Hymenoptera	Andrenidae	*Andrena discors*
Hymenoptera	Andrenidae	*Andrena flavipes*
Hymenoptera	Andrenidae	*Andrena pilipes*
Hymenoptera	Andrenidae	*Andrena* sp1
Hymenoptera	Andrenidae	*Andrena trimmerana*
Hymenoptera	Megachilidae	*Anthidiellum strigatum*
Hymenoptera	Megachilidae	*Anthidium manicatum*
Diptera	Anthomyiidae	*Anthomyia pluvialis*
Diptera	Anthomyiidae	*Anthomyia* sp1
Diptera	Anthomyiidae	Anthomyiidae sp1
Diptera	Anthomyiidae	Anthomyiidae sp2
Diptera	Anthomyiidae	Anthomyiidae sp3
Hymenoptera	Apidae	*Anthophora atroalba*
Hymenoptera	Apidae	*Anthophora bimaculata*
Hymenoptera	Apidae	*Anthophora plumipes*
Diptera	Bombyliidae	*Anthrax trifasciatus*
Hymenoptera	Apidae	*Apis mellifera*
Lepidoptera	Lycaenidae	*Aricia cramera*
Hymenoptera	unidentified	Bee sp1
Hymenoptera	unidentified	Bee sp2
Hymenoptera	unidentified	Bee sp3
Hymenoptera	Crabronidae	*Bembix* sp1
Hymenoptera	Apidae	*Bombus terrestris*
Diptera	Bombyliidae	Bombyliidae sp1
Diptera	Bombyliidae	*Bombylius cinerascens*
Diptera	Bombyliidae	*Bombylius medius*
Lepidoptera	Lycaenidae	*Cacyreus marshalli*
Diptera	Calliphoridae	*Calliphora vicina*
Diptera	Calliphoridae	*Calliphora vomitoria*
Hymenoptera	Apidae	*Ceratina cucurbitina*
Hymenoptera	Apidae	*Ceratina dentiventris*
Hymenoptera	Apidae	*Ceratina saundersi*
Hymenoptera	Crabronidae	*Cerceris sabulosa*
Hymenoptera	Halictidae	*Ceylalictus variegatus*
Hymenoptera	Megachilidae	*Chelostoma florisomne*
Hymenoptera	Chrysididae	*Chrysis ignita*
Hymenoptera	Chrysididae	*Chrysis* sp1
Diptera	Syrphidae	*Chrysotoxum intermedium*
Hymenoptera	Megachilidae	*Coelioxys aurolimbatus*
Coleoptera	unidentified	Coleoptera sp1
Hymenoptera	Colletidae	*Colletes* sp1
Hymenoptera	Crabronidae	Crabronidae sp1
Hymenoptera	Scoliidae	*Dasyscolia ciliata*
Diptera	Syrphidae	*Episyrphus balteatus*
Diptera	Syrphidae	*Eristalinus aeneus*
Diptera	Syrphidae	*Eristalinus taeniops*
Diptera	Syrphidae	*Eristalis arbustorum*

Order	Family	Species name
Diptera	Syrphidae	*Eristalis similis*
Diptera	Syrphidae	*Eristalis tenax*
Hymenoptera	Apidae	*Eucera collaris*
Hymenoptera	Apidae	*Eucera elongatula*
Hymenoptera	Apidae	*Eucera notata*
Hymenoptera	Vespidae	*Euodynerus* sp1
Diptera	Syrphidae	*Eupeodes corollae*
Diptera	unidentified	Fly sp1
Diptera	unidentified	Fly sp2
Diptera	Muscidae	*Graphomya maculata*
Hymenoptera	Halictidae	*Halictus fulvipes*
Hymenoptera	Halictidae	*Halictus scabiosae*
Hymenoptera	Halictidae	*Halictus sexcinctus*
Hymenoptera	Halictidae	*Halictus* sp1
Hymenoptera	Halictidae	*Halictus vestitus*
Diptera	Muscidae	*Helina evecta*
Coleoptera	Tenebrionidae	*Heliotaurus ruficollis*
Hymenoptera	Megachilidae	*Heriades crenulatus*
Hymenoptera	Megachilidae	*Hoplitis adunca*
Hymenoptera	Megachilidae	*Hoplitis benoisti*
Hymenoptera	Megachilidae	*Hoplitis papaveri*
Diptera	Syrphidae	Hoverfly sp1
Diptera	Syrphidae	Hoverfly sp2
Hymenoptera	Colletidae	*Hylaeus pictipes*
Hymenoptera	Colletidae	*Hylaeus pictus*
Hymenoptera	Colletidae	*Hylaeus punctatus*
Hymenoptera	Colletidae	*Hylaeus* sp1
Hymenoptera	Ichneumonidae	Ichneumonidae sp1
Lepidoptera	Lycaenidae	*Lampides boeticus*
Hymenoptera	Halictidae	*Lasioglossum algericolellum*
Hymenoptera	Halictidae	*Lasioglossum capitale*
Hymenoptera	Halictidae	*Lasioglossum costulatum*
Hymenoptera	Halictidae	*Lasioglossum glabriusculum*
Hymenoptera	Halictidae	*Lasioglossum leucozonium cedri*
Hymenoptera	Halictidae	*Lasioglossum malachurum*
Hymenoptera	Halictidae	*Lasioglossum minutissimum*
Hymenoptera	Halictidae	*Lasioglossum pauperatum*
Hymenoptera	Halictidae	*Lasioglossum pauxillum*
Hymenoptera	Halictidae	*Lasioglossum politum*
Hymenoptera	Halictidae	*Lasioglossum* sp1
Hymenoptera	Halictidae	*Lasioglossum sphecodimorphum*
Lepidoptera	unidentified	Lepidoptera sp1
Lepidoptera	unidentified	Lepidoptera sp2
Lepidoptera	Lycaenidae	*Leptotes pirithous*
Hymenoptera	Leucospidae	*Leucospis* sp1
Diptera	Calliphoridae	*Lucilia sericata*
Diptera	Calliphoridae	*Lucilia* sp1
Diptera	Calliphoridae	*Lucilia* sp2
Lepidoptera	Lycaenidae	*Lycaena phlaeas*
Lepidoptera	Sphingidae	*Macroglossum stellatarum*
Hymenoptera	Megachilidae	*Megachile apicalis*
Hymenoptera	Megachilidae	*Megachile ericetorum*
Hymenoptera	Megachilidae	*Megachile pilidens*
Hymenoptera	Megachilidae	*Megachile rotundata*
Hymenoptera	Megachilidae	*Megachile* sp1

Order	Family	Species name
Hymenoptera	Megachilidae	*Megachile willughbiella*
Diptera	Syrphidae	*Melanostoma mellinum*
Diptera	Syrphidae	*Merodon geniculatus*
Diptera	unidentified	Morphospecies18
Diptera	unidentified	Morphospecies3
Diptera	unidentified	Mosca sp1
Diptera	Muscidae	*Musca autumnalis*
Diptera	Muscidae	*Musca domestica*
Diptera	Muscidae	Muscidae sp1
Diptera	Muscidae	Muscidae sp2
Diptera	Muscidae	Muscidae sp3
Diptera	Muscidae	*Muscina stabulans*
Diptera	Syrphidae	*Myathropa florea*
Diptera	Syrphidae	*Myolepta difformis*
Hymenoptera	Apidae	*Nomada gribodoi*
Hymenoptera	Apidae	*Nomada merceti*
Hymenoptera	Apidae	*Nomada numida*
Hymenoptera	Apidae	*Nomada* sp1
Hymenoptera	Apidae	*Nomada succincta*
Hymenoptera	Halictidae	*Nomiapis bispinosa*
Hymenoptera	Apidae	*Nomioides facilis*
Hymenoptera	Megachilidae	*Osmia andrenoides*
Hymenoptera	Megachilidae	*Osmia bicornis*
Hymenoptera	Megachilidae	*Osmia caerulescens*
Hymenoptera	Megachilidae	*Osmia* sp1
Hymenoptera	Megachilidae	*Osmia submicans*
Coleoptera	Cetoniidae	*Oxythyrea funesta*
Hymenoptera	Andrenidae	*Panurgus canescens*
Lepidoptera	Papilionidae	*Papilio machaon*
Diptera	Syrphidae	*Paragus bicolor*
Diptera	Syrphidae	*Paragus* sp1
Diptera	Syrphidae	*Paragus quadrifasciatus*
Lepidoptera	Pieridae	*Pieris rapae*
Hymenoptera	Sphecidae	*Podalonia* sp1
Hymenoptera	Vespidae	*Polistes gallicus*
Diptera	Calliphoridae	*Pollenia amentaria*
Diptera	Calliphoridae	*Pollenia* sp1
Diptera	Calliphoridae	*Pollenia* sp2
Coleoptera	Scarabaeidae	*Protaetia* sp1
Coleoptera	Cantharidae	*Rhagonycha fulva*
Hymenoptera	Megachilidae	*Rhodanthidium sticticum*
Hymenoptera	Vespidae	*Rhynchium oculatum*
Diptera	Sarcophagidae	*Sarcophaga* sp1
Hymenoptera	Sphecidae	*Sceliphron* sp1
Hymenoptera	Halictidae	*Seladonia confusa perkinsi*
Hymenoptera	Halictidae	*Seladonia gemmea*
Hymenoptera	Halictidae	*Seladonia smaragdula*
Hymenoptera	Crabronidae	*Solierella* sp1
Diptera	Syrphidae	*Sphaerophoria rueppellii*
Diptera	Syrphidae	*Sphaerophoria scripta*
Hymenoptera	Megachilidae	*Stelis punctulatissima*
Diptera	Rhinophoridae	*Stevenia* sp1
Diptera	Calliphoridae	*Stomorhina lunata*
Diptera	Syrphidae	*Syritta flaviventris*
Diptera	Syrphidae	*Syritta pipiens*

Order	Family	Species name
Hymenoptera	Apidae	*Thyreus histrionicus*
Lepidoptera	Nymphalidae	*Vanessa atalanta*
Lepidoptera	Nymphalidae	*Vanessa cardui*
Hymenoptera	Vespidae	Vespidae sp1
Hymenoptera	Vespidae	*Vespula* sp1
Hymenoptera	Halictidae	*Vestitohalictus pollinosus*
Diptera	Bombyliidae	*Villa* sp1
Diptera	Syrphidae	*Xanthandrus comtus*
Hymenoptera	Apidae	*Xylocopa violacea*
