# Supplementary material for: Non-native plant integration into plant-insect pollinator networks in urban parks
Source: PLoS One. 2026 Jul 14;21(7):e0353207. doi: 10.1371/journal.pone.0353207 (PMC13367714; doi:10.1371/journal.pone.0353207)
Supplement: S4 Table — (PDF) [file pone.0353207.s004.pdf]

Table S4. Sampling completeness of interaction diversity in census of each park in each period and in total.

Park	Period 1	Period 2	Period 3	Period 4	Total
Alamillo	0.70	0.70	0.67	0.58	0.66
Álvaro Diamantino Vellisco	0.74	0.42	0.73	-	0.67
Amate	0.57	0.61	0.65	0.76	0.57
Bermejales	0.38	0.77	0.78	0.71	0.62
José Celestino Mutis	0.61	0.79	0.33	0.37	0.61
Federico García Lorca	0.64	0.69	0.24	0.29	0.62
Infanta Elena	0.75	0.50	0.73	0.78	0.63
Jardines de la Buhaira	0.75	0.69	0.43	0.53	0.71
Jardines del Guadalquivir	0.62	0.69	0.32	0.28	0.68
Jardines del Valle	0.57	0.69	0.79	0.39	0.64
Jose María de los Santos	0.52	0.84	0.80	0.20	0.55
María Luisa	0.80	0.59	0.90	0.55	0.69
Don Miguel Mañara	0.74	1.00	1.00	-	0.74
Parque de los Príncipes	0.68	0.69	0.85	0.53	0.75
Tamarguillo	0.49	0.46	0.62	0.82	0.56
