# Supplementary material for: Non-native plant integration into plant-insect pollinator networks in urban parks
Source: PLoS One. 2026 Jul 14;21(7):e0353207. doi: 10.1371/journal.pone.0353207 (PMC13367714; doi:10.1371/journal.pone.0353207)
Supplement: S5 Table — (PDF) [file pone.0353207.s005.pdf]

Table S5. Modularity characteristics of each park.

Park	Value	z-value	p-value	Number of modules	Mean number of species by module (plants/pollinators)	Mean % non-native plant taxa by module
Alamillo	0.3170	20.40	<0.001	6	2.33 / 8.17	55.56
Álvaro Diamantino Vellisco	0.3584	7.86	<0.001	5	1.20 / 4.00	100
Amate	0.3982	33.46	<0.001	6	4.67 / 9.50	88.89
Bermejales	0.3587	24.83	<0.001	5	2.00 / 10.20	50
José Celestino Mutis	0.4054	30.14	<0.001	9	3.11 / 4.44	93.15
Federico García Lorca	0.3484	16.77	<0.001	4	2.50 / 9.25	100
Infante Elena	0.5322	28.06	<0.001	7	2.43 / 7.14	50.83
Jardines de la Buhaira	0.4130	20.25	<0.001	5	2.20 / 7.40	93.33
Jardines del Guadalquivir	0.4597	33.25	<0.001	8	3.38 / 8.00	81.88
Jardines del Valle	0.3920	30.19	<0.001	4	4.00 / 11.00	93.75
Jose María de los Santos	0.4443	25.48	<0.001	6	2.00 / 7.50	88.33
María Luisa	0.4266	37.19	<0.001	6	4.33 / 7.50	86.11
Don Miguel Mañara	0.2616	6.80	<0.001	3	1.33 / 4.33	100
Parque de los Príncipes	0.3881	37.39	<0.001	6	4.17 / 8.67	94.84
Tamarguillo	0.4307	33.97	<0.001	8	2.38 / 8.25	71.43
