# Supplementary material for: Non-native plant integration into plant-insect pollinator networks in urban parks
Source: PLoS One. 2026 Jul 14;21(7):e0353207. doi: 10.1371/journal.pone.0353207 (PMC13367714; doi:10.1371/journal.pone.0353207)
Supplement: S6 Table — Best GLMM is highlighted in bold by each speces-level network parameter. When more than one model were the best, we showed in results the model which includes plant origin (for within-module degree, z). (PDF) [file pone.0353207.s006.pdf]

Table S6. Summary of generalized linear mixed models (GLMMs) with species-level network parameters against plant taxa origin, period, floral availability, network size, and flowering length. Best GLMM is highlighted in bold by each speces-level network parameter. When more than one model were the best, we showed in results the model which includes plant origin (for within-module degree, *z*).

Parameter	Model	df	AIC	ΔAIC
Normalized degree	Origin + (1|Park)	4	840.09	110.64
	Period + (1|Park)	6	807.56	78.11
	scale(Floral availability) + (1|Park)	4	810.29	80.84
	Network_size + (1|Park)	4	779.84	50.39
	Origin + Period + (1|Park)	7	811.98	82.53
	Origin + scale(Floral availability) + (1|Park)	5	815.01	85.56
	Origin + Network_size + (1|Park)	5	785.22	55.77
	Period + scale(Floral availability) + (1|Park)	7	754.10	24.65
	Period + Network_size + (1|Park)	7	790.78	61.33
	Origin + Period + Network_size + (1|Park)	8	796.17	66.72
	Origin + Period + scale(Floral availability) + (1|Park)	8	759.25	29.80
	Origin + Period + scale(Floral availability) + Network_size + (1|Park)	9	734.88	5.43
	**Period + scale(Floral availability) + Network_size + (1|Park)**	**8**	**729.45**	**0**
	Origin * Period + (1|Park)	10	821.55	92.10
	Origin * scale(Floral availability) + (1|Park)	6	812.30	82.85
	Origin * Network_size + (1|Park)	6	795.51	66.06
	Period * scale(Floral availability) + (1|Park)	10	756.50	27.05
	Period * Network_size + (1|Park)	10	813.77	84.32
	Origin * Period + scale(Floral availability) + Network_size + (1|Park)	12	744.26	14.81
	Origin + Period * scale(Floral availability) + Network_size + (1|Park)	12	734.66	5.21
	Origin + Period + scale(Floral availability) * Network_size + (1|Park)	10	743.48	14.03
	Origin * Period * scale(Floral availability) * Network_size + (1|Park)	34	843.91	114.46
Species strength	Origin + (1|Park)	4	1,123.16	61.56
	Period + (1|Park)	6	1,103.76	42.16
	scale(Floral availability) + (1|Park)	4	1,125.77	64.17
	Network_size + (1|Park)	4	1,070.65	9.05
	Origin + Period + (1|Park)	7	1,100.61	39.01
	Origin + scale(Floral availability) + (1|Park)	5	1,123.35	61.75
	Origin + Network_size + (1|Park)	5	1,065.80	4.20
	Period + scale(Floral availability) + (1|Park)	7	1,092.18	30.58
	Period + Network_size + (1|Park)	7	1,080.02	18.42
	Origin + Period + Network_size + (1|Park)	8	1,075.08	13.48
	Origin + Period + scale(Floral availability) + (1|Park)	8	1,090.49	28.89
	**Origin + Period + scale(Floral availability) + Network_size + (1|Park)**	**9**	**1,061.60**	**0**
	Period + scale(Floral availability) + Network_size + (1|Park)	8	1,065.16	3.56
	Origin * Period + (1|Park)	10	1,105.76	44.16
	Origin * scale(Floral availability) + (1|Park)	6	1,127.37	65.77
	Origin * Network_size + (1|Park)	6	1,075.32	13.72
	Period * scale(Floral availability) + (1|Park)	10	1,099.97	38.37
	Period * Network_size + (1|Park)	10	1,101.70	40.10
	Origin * Period + scale(Floral availability) + Network_size + (1|Park)	12	1,066.21	4.61
	Origin + Period * scale(Floral availability) + Network_size + (1|Park)	12	1,068.48	6.88
	Origin + Period + scale(Floral availability) * Network_size + (1|Park)	10	1,072.56	10.96
	Origin * Period * scale(Floral availability) * Network_size + (1|Park)	34	1,163.21	101.61
Contribution to nestedness	Origin + (1|Park)	4	1,120.22	43.77
	Period + (1|Park)	6	1,107.47	31.02
	scale(Floral availability) + (1|Park)	4	1,080.48	4.03
	Network_size + (1|Park)	4	1,105.50	29.05

	Origin + Period + (1|Park)	7	1,111.54	35.09
	Origin + scale(Floral availability) + (1|Park)	5	1,084.89	8.44
	Origin + Network_size + (1|Park)	5	1,109.93	33.48
	Period + scale(Floral availability) + (1|Park)	7	1,079.51	3.06
	Period + Network_size + (1|Park)	7	1,103.47	27.02
	Origin + Period + Network_size + (1|Park)	8	1,107.95	31.5
	Origin + Period + scale(Floral availability) + (1|Park)	8	1,083.94	7.49
	Origin + Period + scale(Floral availability) + Network_size + (1|Park)	9	1,080.81	4.36
	**Period + scale(Floral availability) + Network_size + (1|Park)**	**8**	**1,076.45**	**0**
	Origin * Period + (1|Park)	10	1,118.30	41.85
	Origin * scale(Floral availability) + (1|Park)	6	1,087.44	10.99
	Origin * Network_size + (1|Park)	6	1,118.98	42.53
	Period * scale(Floral availability) + (1|Park)	10	1,084.72	8.27
	Period * Network_size + (1|Park)	10	1,123.06	46.61
	Origin * Period + scale(Floral availability) + Network_size + (1|Park)	12	1,083.94	7.49
	Origin + Period * scale(Floral availability) + Network_size + (1|Park)	12	1,087.37	10.92
	Origin + Period + scale(Floral availability) * Network_size + (1|Park)	10	1,092.02	15.57
	Origin * Period * scale(Floral availability) * Network_size + (1|Park)	34	1,167.08	90.63
Specialization (d')	Origin + (1|Park)	4	-24.85	4.73
	Period + (1|Park)	6	-27.33	2.25
	scale(Floral availability) + (1|Park)	4	-23.48	6.10
	Network_size + (1|Park)	4	-18.91	10.67
	Origin + Period + (1|Park)	7	-20.27	9.31
	Origin + scale(Floral availability) + (1|Park)	5	-16.43	13.15
	Origin + Network_size + (1|Park)	5	-11.76	17.82
	Period + scale(Floral availability) + (1|Park)	7	-19.09	10.49
	Period + Network_size + (1|Park)	7	-18.76	10.82
	Origin + Period + Network_size + (1|Park)	8	-11.54	18.04
	Origin + Period + scale(Floral availability) + (1|Park)	8	-12.11	17.47
	Origin + Period + scale(Floral availability) + Network_size + (1|Park)	9	-3.48	26.10
	Period + scale(Floral availability) + Network_size + (1|Park)	8	-10.65	18.93
	**Origin * Period + (1|Park)**	**10**	**-29.58**	**0**
	Origin * scale(Floral availability) + (1|Park)	6	-13.05	16.53
	Origin * Network_size + (1|Park)	6	-1.99	27.59
	Period * scale(Floral availability) + (1|Park)	10	-7.32	22.26
	Period * Network_size + (1|Park)	10	2.69	32.27
	Origin * Period + scale(Floral availability) + Network_size + (1|Park)	12	-11.31	18.27
	Origin + Period * scale(Floral availability) + Network_size + (1|Park)	12	7.92	37.5
	Origin + Period + scale(Floral availability) * Network_size + (1|Park)	10	8.74	38.32
	Origin * Period * scale(Floral availability) * Network_size + (1|Park)	34	119.55	149.13
within-module degree, *z*	Origin + (1|Park)	4	589.88	49.05
	Flowering length + (1|Park)	**4**	**540.83**	**0**
	scale(Floral availability) + (1|Park)	4	579.81	38.98
	Network_size + (1|Park)	4	601.96	61.13
	**Origin + Flowering length + (1|Park)**	**5**	**541.54**	**0.71**
	Origin + scale(Floral availability) + (1|Park)	5	580.28	39.45
	Origin + Network_size + (1|Park)	5	601.48	60.65
	Flowering length + scale(Floral availability) + (1|Park)	5	546.57	5.74
	Flowering length + Network_size + (1|Park)	5	552.65	11.82
	Origin + Flowering length + Network_size + (1|Park)	6	553.40	12.57
	Origin + Flowering length + scale(Floral availability) + (1|Park)	6	547.36	6.53
	Origin + Flowering length + scale(Floral availability) + Network_size + (1|Park)	7	559.22	18.39
	Flowering length + scale(Floral availability) + Network_size + (1|Park)	6	558.39	17.56
	Origin * Flowering length + (1|Park)	6	543.54	2.71
	Origin * scale(Floral availability) + (1|Park)	6	584.19	43.36
	Origin * Network_size + (1|Park)	6	609.42	68.59

	Flowering length * scale(Floral availability) + (1|Park)	6	549.33	8.50
	Flowering length * Network_size + (1|Park)	6	560.04	19.21
	Origin * Flowering length + scale(Floral availability) + Network_size + (1|Park)	8	560.91	20.08
	Origin + Flowering length * scale(Floral availability) + Network_size + (1|Park)	8	563.34	22.51
	Origin + Flowering length + scale(Floral availability) * Network_size + (1|Park)	8	569.17	28.34
	Origin * Flowering length * scale(Floral availability) * Network_size + (1|Park)	18	630.18	89.35
among-module connectivity, *c*	Origin + (1|Park)	4	-292.68	52.20
	Flowering length + (1|Park)	4	-328.72	16.16
	scale(Floral availability) + (1|Park)	4	-300.75	44.13
	Network_size + (1|Park)	4	-297.75	47.13
	Origin + Flowering length + (1|Park)	5	-326.94	17.94
	Origin + scale(Floral availability) + (1|Park)	5	-298.99	45.89
	Origin + Network_size + (1|Park)	5	-296.11	48.77
	Flowering length + scale(Floral availability) + (1|Park)	5	-327.00	17.88
	Flowering length + Network_size + (1|Park)	5	-332.29	12.59
	Origin + Flowering length + Network_size + (1|Park)	6	-331.26	13.62
	Origin + Flowering length + scale(Floral availability) + (1|Park)	6	-325.18	19.70
	Origin + Flowering length + scale(Floral availability) + Network_size + (1|Park)	7	-329.45	15.43
	Flowering length + scale(Floral availability) + Network_size + (1|Park)	6	-330.57	14.31
	Origin * Flowering length + (1|Park)	6	-327.92	16.96
	Origin * scale(Floral availability) + (1|Park)	6	-297.01	47.87
	Origin * Network_size + (1|Park)	6	-295.32	49.56
	Flowering length * scale(Floral availability) + (1|Park)	6	-340.55	4.33
	Flowering length * Network_size + (1|Park)	6	-331.00	13.88
	Origin * Flowering length + scale(Floral availability) + Network_size + (1|Park)	8	-330.63	14.25
	**Origin + Flowering length * scale(Floral availability) + Network_size + (1|Park)**	**8**	**-344.88**	**0**
	Origin + Flowering length + scale(Floral availability) * Network_size + (1|Park)	8	-327.52	17.36
	Origin * Flowering length * scale(Floral availability) * Network_size + (1|Park)	18	-335.14	9.74
