# Supplementary material for: Non-native plant integration into plant-insect pollinator networks in urban parks
Source: PLoS One. 2026 Jul 14;21(7):e0353207. doi: 10.1371/journal.pone.0353207 (PMC13367714; doi:10.1371/journal.pone.0353207)
Supplement: S7 Table — Statistically significant terms are highlighted in bold. (PDF) [file pone.0353207.s007.pdf]

Table S7. Summary of generalized linear mixed model (GLMM) with ‘number of periods in which each plant-plant pair co-flowered’ as the dependent variable. Statistically significant terms are highlighted in bold.

**Coefficient**	**Value**	**Standard error**	**z-value**	**p-value**
Intercept	-0.016	0.112	-0.141	0.888
Native-native	-0.214	0.257	-0.834	0.404
**Nonnative-nonnative**	**-0.342**	**0.136**	**-2.527**	**0.011**
