# Supplementary material for: Non-native plant integration into plant-insect pollinator networks in urban parks
Source: PLoS One. 2026 Jul 14;21(7):e0353207. doi: 10.1371/journal.pone.0353207 (PMC13367714; doi:10.1371/journal.pone.0353207)
Supplement: S1 Fig — Censused months where grouped according to their similarity in flowering species, obtaining four separate clusters (periods). (PDF) [file pone.0353207.s008.pdf]

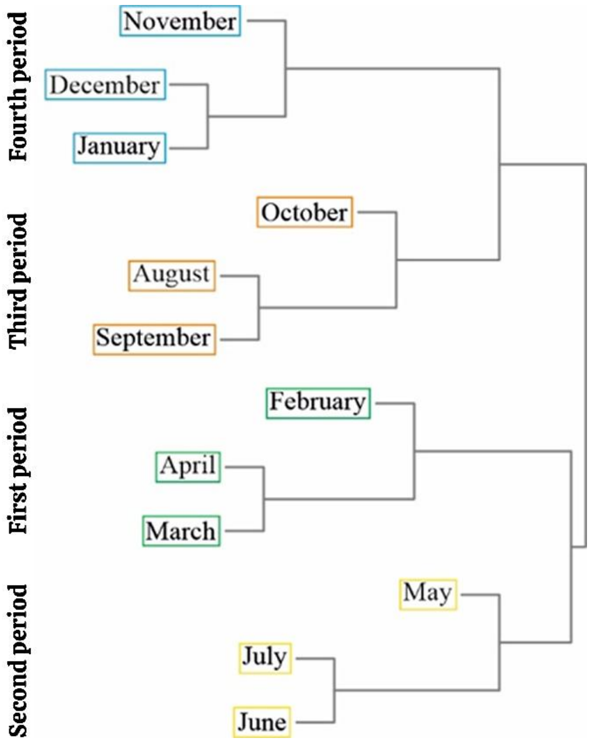

Figure S1. Dendogram showing flowering plant species phenology richness similarity (Jaccard similarity matrix) among months. Censused months where grouped according to their similarity in flowering species, obtaining four separate clusters (periods).
