# Supplementary material for: Non-native plant integration into plant-insect pollinator networks in urban parks
Source: PLoS One. 2026 Jul 14;21(7):e0353207. doi: 10.1371/journal.pone.0353207 (PMC13367714; doi:10.1371/journal.pone.0353207)
Supplement: S2 Fig — Data were pooled across all parks for visual clarity. Each subpanel represents each period (Period 1: February-March; Period 2: May-July; Period 3: August-October; Period 4: November-January). (PDF) [file pone.0353207.s009.pdf]

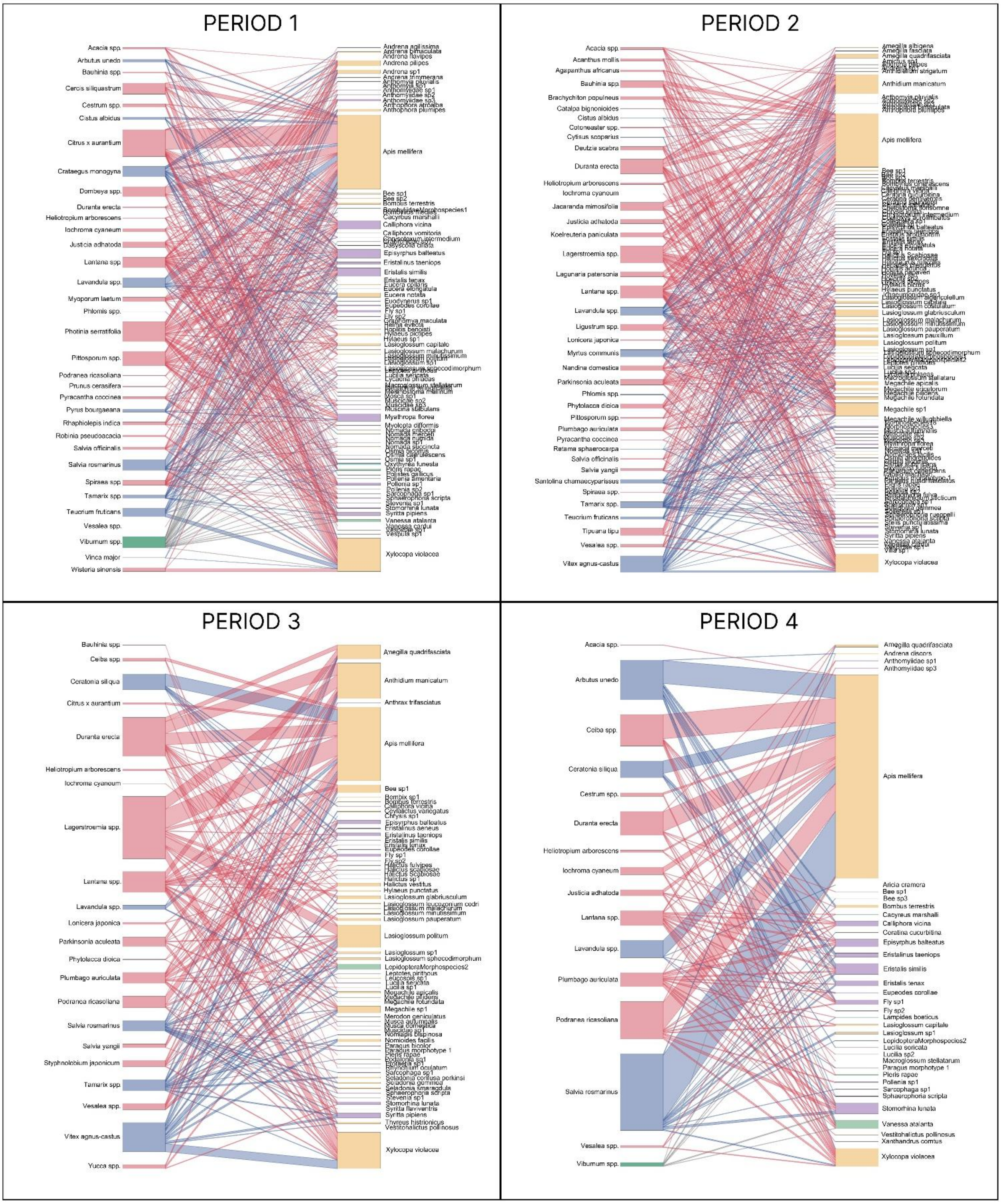

Figure S2. Representation of plant-pollinator networks over periods. Data were pooled across all parks for visual clarity. Each subpanel represents each period (Period 1: February-March; Period 2: May-July; Period 3: August-October; Period 4: November-January). Colours for plant taxa represent their plant origin (red: non-native, blue: native, green: *Viburnum* spp.). Colours for pollinators taxa represents their order (yellow: Hymenoptera, green: Lepidoptera, purple: Diptera, turquoise: Coleoptera).
