# Supplementary material for: Non-native plant integration into plant-insect pollinator networks in urban parks
Source: PLoS One. 2026 Jul 14;21(7):e0353207. doi: 10.1371/journal.pone.0353207 (PMC13367714; doi:10.1371/journal.pone.0353207)
Supplement: S3 Fig — Black boxes represent modules. Darker colors correspond to higher frequency of interactions. Red tones correspond to non-native plant taxa, blue tones, to native plant taxa and green tones to Viburnum spp. A: Alamillo, B: Álvaro Diamantino Vellisco, C: Amate, D: Los Bermejales, E: José Celestino Mutis, F: Federico García Lorca, G: Infanta Elena, H: Jardines de la Buhaira, I: Jardines del Guadalquivir, J: Jardines del Valle, K: José María de los Santos, L: Maria Luisa, M: Don Miguel Mañara, N: Parque de los Príncipes, O: Tamarguillo. (PDF) [file pone.0353207.s010.pdf]

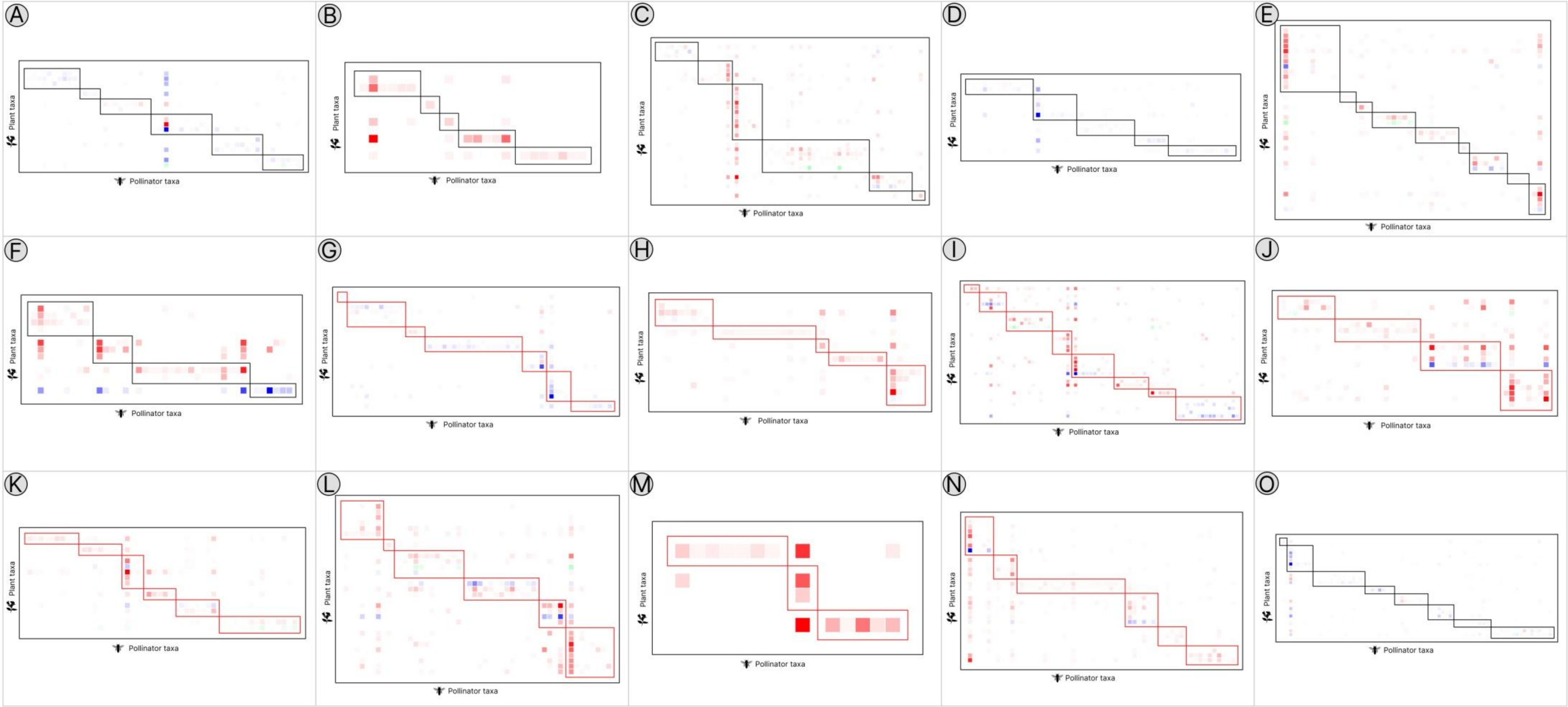

Figure S3. Modularity *M* of each park. Black boxes represent modules. Darker colors correspond to higher frequency of interactions. Red tones correspond to non-native plant taxa, blue tones, to native plant taxa and green tones to *Viburnum* spp. A: Alamillo, B: Álvaro Diamantino Vellisco, C: Amate, D: Los Bermejales, E: José Celestino Mutis, F: Federico García Lorca, G: Infanta Elena, H: Jardines de la Buhaira, I: Jardines del

Guadalquivir, J: Jardines del Valle, K: José María de los Santos, L: Maria Luisa, M: Don Miguel Mañara, N: Parque de los Príncipes, O: Tamarguillo.
