# Supplementary material for: Non-native plant integration into plant-insect pollinator networks in urban parks
Source: PLoS One. 2026 Jul 14;21(7):e0353207. doi: 10.1371/journal.pone.0353207 (PMC13367714; doi:10.1371/journal.pone.0353207)
Supplement: S5 Fig — (PDF) [file pone.0353207.s012.pdf]

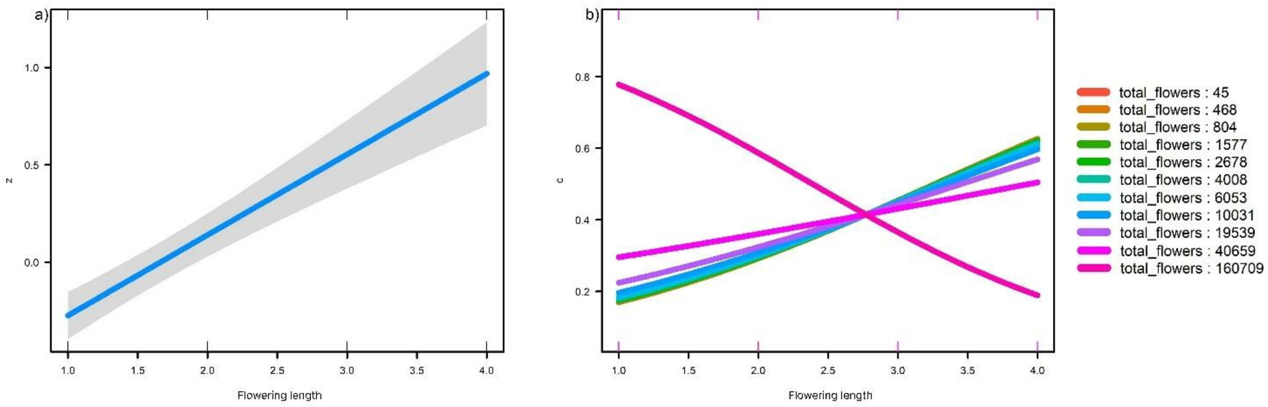

Figure S5. Regression model fits for a) within-module degree z against flowering length and b) among-module connectivity c against the interaction between flowering length using *visreg* R package (Breheny & Burchett, 2017).

Reference

Breheny, P. & Burchett, W. (2017). Visualization of Regression Models Using visreg. *The R journal*, 9(2), 56-71.
