# Supplementary material for: Non-native plant integration into plant-insect pollinator networks in urban parks
Source: PLoS One. 2026 Jul 14;21(7):e0353207. doi: 10.1371/journal.pone.0353207 (PMC13367714; doi:10.1371/journal.pone.0353207)
Supplement: S6 Fig — Circles are plant-plant pairs that co-flowering in each period. Colours show the plant taxa origin: yellow: mix (one native and one non-native), red: non-native (both non-native) and blue native (both native). A: Alamillo, B: Álvaro Diamantino Vellisco, C: Amate, D: Los Bermejales, E: José Celestino Mutis, F: Federico García Lorca, G: Infanta Elena, H: Jardines de la Buhaira, I: Jardines del Guadalquivir, J: Jardines del Valle, K: José María de los Santos, L: Maria Luisa, M: Don Miguel Mañara, N: Parque de los Príncipes, O: Tamarguillo. (PDF) [file pone.0353207.s013.pdf]

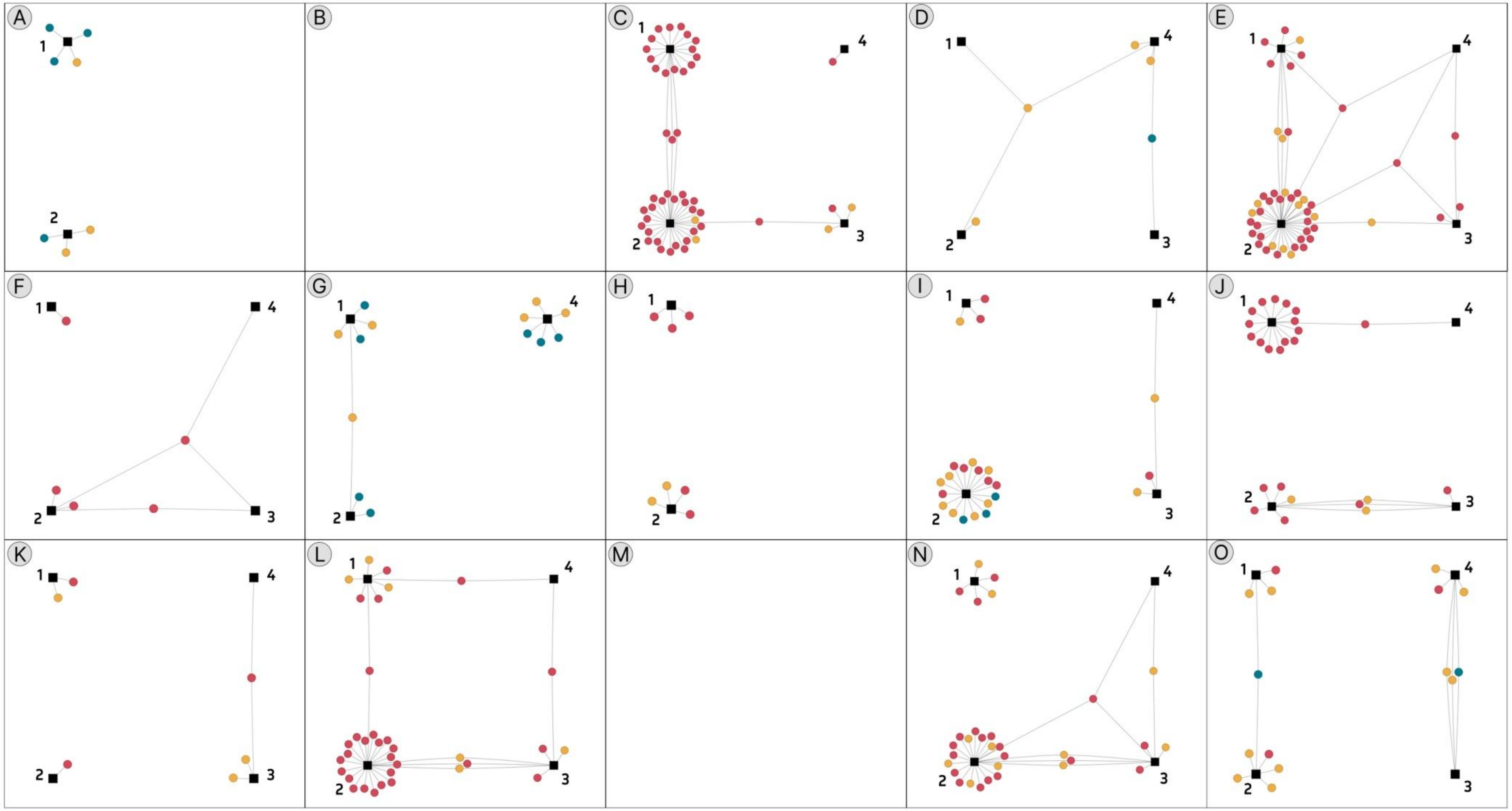

Figure S6. Representation of co-flowering of plants that form modules together throughout the four temporal periods (from 1 to 4) for each park. Circles are plant-plant pairs that co-flowering in each period. Colours show the plant taxa origin: yellow: mix (one native and one non-native), red: non-native (both non-native) and blue native (both native). A: Alamillo, B: Álvaro Diamantino Vellisco, C: Amate, D: Los Bermejales, E: José

Celestino Mutis, F: Federico García Lorca, G: Infanta Elena, H: Jardines de la Buhaira, I: Jardines del Guadalquivir, J: Jardines del Valle, K: José María de los Santos, L: Maria Luisa, M: Don Miguel Mañara, N: Parque de los Príncipes, O: Tamarguillo.
