# Supplementary material for: Non-native plant integration into plant-insect pollinator networks in urban parks
Source: PLoS One. 2026 Jul 14;21(7):e0353207. doi: 10.1371/journal.pone.0353207 (PMC13367714; doi:10.1371/journal.pone.0353207)
Supplement: S7 Fig — Black squares denote the four temporal sampling periods (1–4). Each circle represents a unique pairwise interaction between a plant taxon and a pollinator taxon in a single period, while triangles represent interactions shared among periods. Colors indicate the origin of the plant taxon involved in each interaction (red: non-native, blue: native). Grey lines connect interactions to the periods in which they were recorded. Each subpanel represents a park: A: Alamillo, B: Álvaro Diamantino Vellisco, C: Amate, D: Los Bermejales, E: José Celestino Mutis, F: Federico García Lorca, G: Infanta Elena, H: Jardines de la Buhaira, I: Jardines del Guadalquivir, J: Jardines del Valle, K: José María de los Santos, L: Maria Luisa, M: Don Miguel Mañara, N: Parque de los Príncipes, O: Tamarguillo. Network graphs were built using the visNetwork R package (Almende et al. 2025). (PDF) [file pone.0353207.s014.pdf]

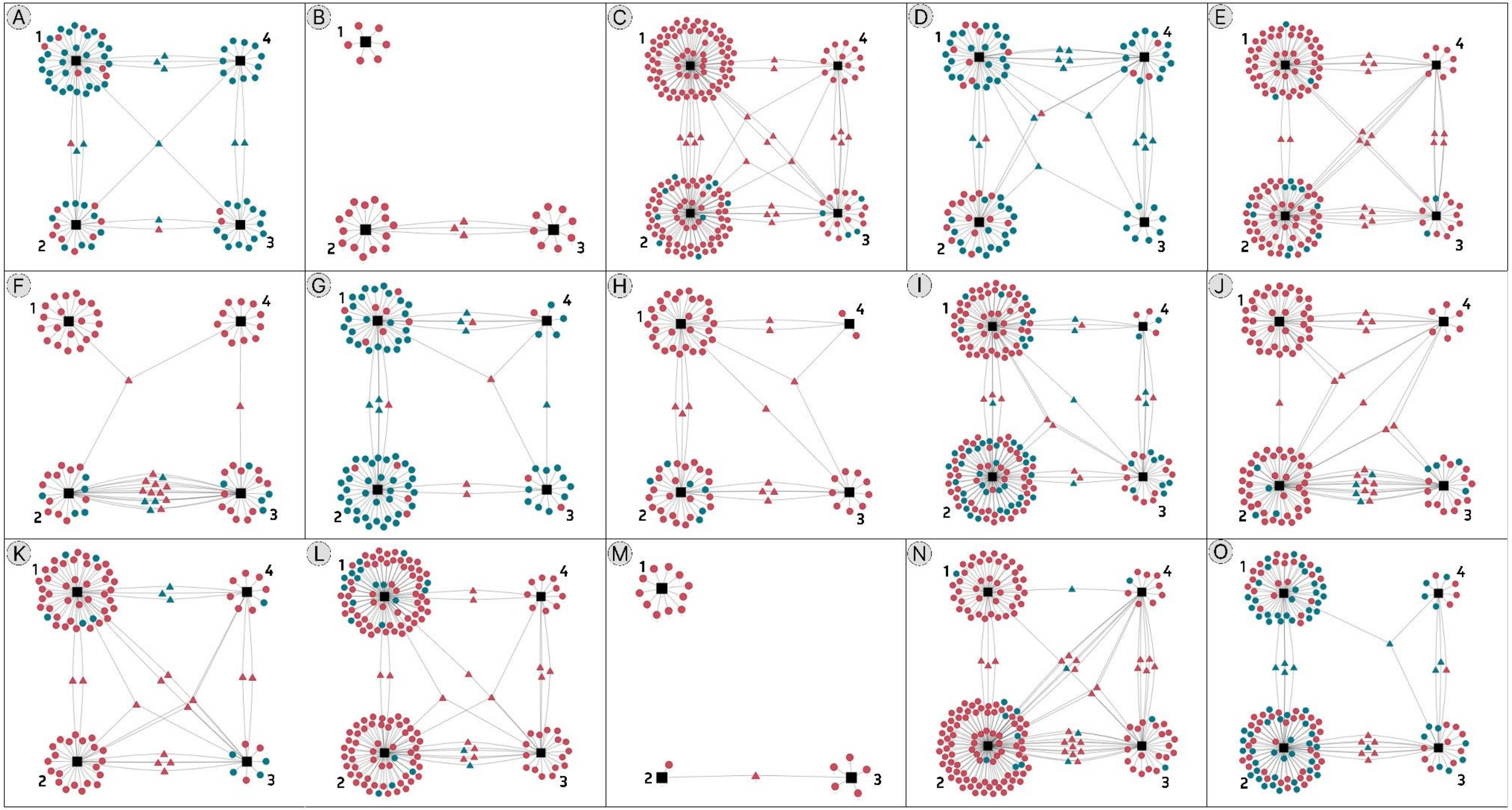

Fig S7. Temporal networks of plant-pollinator interactions across the parks of Seville. Black squares denote the four temporal sampling periods (1-4). Each circle represents a unique pairwise interaction between a plant taxon and a pollinator taxon in a single period, while triangles represent interactions shared among periods. Colors indicate the origin of the plant taxon involved in each interaction (red: non-native, blue: native). Grey

lines connect interactions to the periods in which they were recorded. Each subpanel represents a park: A: Alamillo, B: Álvaro Diamantino Vellisco, C: Amate, D: Los Bermejales, E: José Celestino Mutis, F: Federico García Lorca, G: Infanta Elena, H: Jardines de la Buhaira, I: Jardines del Guadalquivir, J: Jardines del Valle, K: José María de los Santos, L: Maria Luisa, M: Don Miguel Mañara, N: Parque de los Príncipes, O: Tamarguillo. Network graphs were built using the visNetwork R package Almende *et al.* 2025).

Reference

Almende B.V. and Contributors, Thieurmel B (2025). visNetwork: Network Visualization using 'vis.js' Library. R package version 2.1.4, https://github.com/datastorm-open/visnetwork
