# Supplementary material for: Non-native plant integration into plant-insect pollinator networks in urban parks
Source: PLoS One. 2026 Jul 14;21(7):e0353207. doi: 10.1371/journal.pone.0353207 (PMC13367714; doi:10.1371/journal.pone.0353207)
Supplement: S1 Appendix — (PDF) [file pone.0353207.s015.pdf]

Appendix S2.

Sampling design extracted verbatim from Trillo *et al.* (2026).

We selected 15 parks of different sizes (mean = 0.17 km^2, range = 0.003–0.511 km^2), each containing a minimum of four ornamental flowering tree and shrub species. These parks were, on average, 1.7 km apart (range = 0.8–2.5 km), a distance greater than the typical foraging range of most pollinators (Kendall et al., 2022). The parks were located at varying distances from the city edge to the centre (mean = 1.5 km, range = 0.0–3.4 km).

We conducted floral visitor (hereafter, pollinator) censuses in each park over 12 consecutive months (October 2021–September 2022). On average, each park was sampled every 4 weeks, and thus once a month. Between 1 and 6 (mean = 3) randomly selected parks were sampled per day, depending on workload, that is, number of plants surveyed per park. We avoided sampling the same park at the same time slot in consecutive months. In general, the complete monthly sampling of all parks was carried out over the course of one continuous week. Sampling was conducted on sunny, non-windy days, with shade temperatures ranging from 12 to 35°C. All censuses were conducted between 08:45 AM and 7:30 PM.

In each park, we established a fixed 20 m-wide sampling path, which covered the entire area of the park. For the four largest parks (>0.274 km^2), we limited the path to areas with the highest plant diversity, avoiding large extensions of mown areas. The starting point along the sampling path was changed each month. On each sampling day and in each park, we identified all woody ornamental plants in bloom along the path prior to beginning the pollinator censuses. The selected plant species had to meet the following criteria: (a) they were attractive to diurnal pollinators (e.g., we excluded species such as Hibiscus L. (Malvales: Malvaceae), Melia azedarach L. (Sapindales: Meliaceae) and Punica granatum L. (Myrtales: Lythraceae) which are often present in parks but, after a reasonable period of observation (2–3 h), no floral visitors were observed); (b) they were not rare, that is, limited to just a few individuals in a single park; and (c) they allowed for ground-based censuses. Once a plant species met these criteria, we performed two censuses per species, whenever possible at different locations along the sampling path and in a randomised inter-species order to avoid potential biases in temporal and spatial pollinator activity (Zaragoza-Trello et al., 2023). Thereafter, selected species were sampled whenever they were in bloom in any park throughout the year.

During each census, we conducted a 10-min pollinator count per plant, focusing on a 1 m2 area with the highest flower density. We recorded floral visitors that made contact with any reproductive part of a flower, avoiding double counting. Ants, thrips and other flies and beetles smaller than 3 mm were excluded due to rarity on the flowers. Most specimens were identified visually in the field to the lowest possible taxonomic level. A subset of 629 individuals (7.5% of all observed) was captured for identification in the laboratory with the help of an expert (co-author Francisco P. Molina). Specimens were captured using aerial nets, stored in a chilled box, and then frozen at −20°C at the end of each sampling day. All specimens are deposited at the Estación Biológica de Doñana (EBD-CSIC). In total, we conducted 774 censuses (20 min each) over 64 days, amounting to 258 sampling hours.

To test whether the plant species were adequately surveyed, we conducted an additional 30-min consecutive observation each month on a single and randomly selected plant species, focusing on 1 m^2 area. We constructed rarefaction curves and found that, in general, almost no new pollinator species were detected after 20 min of observation.

Finally, for each studied ornamental plant species, we estimated floral availability per plant, day and park, by counting receptive (i.e., pollinator-accessible) flowers in five randomly selected 40 cm^2 areas per plant species, distributed along the fixed path. In addition, using the QGIS programme, we estimated the area occupied by each species in each park by mapping their coverage during walking surveys. Then, for each species, we calculated the average number of flowers per m^2 and multiplied this by the estimated area (in m^2) occupied by the plant in the park. In all cases, the maximum length of the parks, and thus the area where flower estimates were conducted, did not exceed a 500 m buffer radius, a typical area of influence for small pollinators according to their flight foraging distances (Kendall et al., 2022). For plants with very small flowers —Acacia Mill. (Fabales: Fabaceae) and Santolina chamaecyparissus L. (Asterales: Asteraceae)— or bearing aments —C. siliqua, Phytolacca dioica L. (Caryophyllales: Phytolaccaceae) and Tamarix L. (Caryophyllales: Tamaricaceae)— the inflorescence was considered the floral unit.

In total, we sampled 62 plant taxa (74% of which were non-native according to Castroviejo, 2020), with an average of 17 taxa per park (range = 4–29). On average, individual plant taxa were present in 4 parks (range = 1–13). Seventeen plant taxa with cryptic floral traits were grouped at the genus level as there were mixed species and hybrids within and among parks. We assumed these taxa were equally attractive to pollinators.

Reference

Trillo, A., Ragel-Celdrán, D., Molina, F.P. & Vilà, M. (2026). Year-round pollinator visitation of ornamental plants in Mediterranean urban parks. *Insect Conserv. Divers.*, 19, 702–715.
