# Supplementary material for: Non-native plant integration into plant-insect pollinator networks in urban parks
Source: PLoS One. 2026 Jul 14;21(7):e0353207. doi: 10.1371/journal.pone.0353207 (PMC13367714; doi:10.1371/journal.pone.0353207)
Supplement: Attachment — Submitted filename: Comments_PONE-D-25-67039.pdf [file pone.0353207.s016.pdf]

Comments to the MS: PONE-D-25-67039

I was given the manuscript entitled "*Dynamics of non-native plant integration into plant-pollinator networks in urban parks*" The manuscript deals with the role on native and non-native flowering plants in pollination network in Sevilla (Spain). I would point out that without line numbers, the process of reviewing gets incredibly more difficult as I have no means of indicating the point of my comments. I would kindly ask the authors to insert line numbers in the other rounds of revision, thanks.

General opinion: This study aims to understand the temporal dynamics between plants and pollinators in a city over the course of a year, which is a nice new element supporting the study. However, I believe that it cannot be published in its current form because it is too vague in its identification of plants and pollinating insects (there is never a scientific name or reference). How can one discuss a network without taking into account the entities involved? In addition, although the authors should be commended for the amount of analysis they have done, there are some gaps in the presentation and clarity. I would recommend summarising the information and accompanying it with a few informative graphs and tables. The conclusions are very vague; for example, there are no suggestions for urban green management, no examples of "virtuous plants" or "rejected plants" in these networks. In short, the taxonomic and applicative part is practically absent.

I would suggest the authors to include more recent and relevant literature about their topic, especially given the fact that they have included barely any references to other urban plant-pollinator networks in Europe. https://doi.org/10.1111/1365-2435.13642, https://doi.org/10.1016/j.agee.2021.107560, https://doi.org/10.1556/168.2017.18.1.1, https://doi.org/10.1111/1365-2664.14168, https://doi.org/10.1016/j.ufug.2024.128650

There are also other more specific comments, which can be found in the bulleted list below. The authors are free to accept or reject my comments following adequate explanation or justification.

Title and Abstract: The title does not convey the main findings of the study. The abstract is ok.

Introduction: The introduction provides with enough detail to understand the topic of the study. I have only some minor comments.

- When pointing out the hypotheses, I would not separate them from the question, but I would rather continue on the same line. In addition, the questions may also be completely removed to keep only the working hypotheses.
- A key point: in the introduction is not mentioned the focal insect taxa of the study, or all insects? Or all animals?

Methods: The sampling method part needs to be clarified because all the information are randomly placed in the text (or missing) in my opinion. Clearly separate a section for the plant surveys, and a section for pollinator

counting. How were the pollinators identified and counted? At which taxonomic resolution? How the authors dealt with the possibility of pseudo-replication in pollinators?

- 20-m wide or long? Transects are usually 2m wide and 50-100m long
- "for each plant" … species I would guess no?
- How can the authors avoid double counting if they just visually counted the pollinators? How can you distinguish between two individuals of the same species from the same individual? All this part is very confusing to me.
- "We sampled 62 plant taxa" … this is a result, not a pre-requisite (method) of the study.
- If those two parks were rather poor in plant taxa, why just not excluding them? The number of parks would still be high enough for robust analysis.
- Chao1 index: How were the pollinators identified and counted? At which taxonomic resolution? How the authors dealt with the possibility of pseudo-replication in pollinators? Presence (0) – absence (1). Because how can you record abundance if you just visually monitored the insects. Again, are you dealing with insects or all animals?
- Appendix S2: No, I would suggest putting the definition of the metrics here in the main text, they are essential to the readers to understand the study.
- In building GLMMs, have the authors checked for collinearity? Because I would think that in May-August (period) one should also have higher flower abundance; but these two predictors have been included seemingly blindly.
- I understand the usage of AIC, but this comes with a non-negligible problem: different metrics on the same network are analysed in different ways, which can lead to misleading interpretations. Why do not choose a single model that answers all the questions posed by the authors and use it straightforward? This would also simplify the reading and understanding of the results.

Results: The presentation of the results is insufficient. There are no tables... and the text does not even contain the minimum parameters needed to understand the statistical comparisons/models. Everything needs to be formalised. In this form, the results are barely understandable. The more if you consider that the authors have done a lot of analysis, but in this form, everything is very confusing. I suggest the authors reducing and formalising this section, making it more precise. Now there are a lot of statements, supported by supplementary tables that are not immediately readable. The results should be concise, accompanied by a few extremely significant and informative tables and figures and, given that the authors have carried out numerous analyses, divided by "hypothesis" or "question".

- Fig.S2 is almost incomprehensible. First, the four periods are in square 2x2 (which I figured out after a while), second there are no names about the interacting species. At the moment, this figure does not provide relevant information and can therefore be removed or refined.
- Fig.1 please add statistically significant comparisons to the boxplots for all the comparisons OR delete plots showing non-significant comparisons. In addition, where are the tables with the selected models? They were not even mentioned in the text the summary statistics of the models
- Table S4. This table should be placed in the text. Why there is never Period 3? In addition, I think here there is a problem of interpretation. When interactions are statistically significant, they should be discussed as the sole significant terms, and not together with their non-interacting factors. You must

interpret the interaction first. The main effects become less relevant or misleading because the effect of that variable isn't consistent (as suggested by the significant interaction).

- Fig.3 is almost incomprehensible because it does not convey clear information but rather a chaotic representation of the networks though the four seasons. In addition, again, without names it is even less informative.

Discussion: I believe that, in light of what I have read, the entire discussion and study have a major flaw. The authors did not consider the type of plant (the species are never mentioned) or its floral morphology, nor did they consider the type of insect that visited it. How can one generalise without knowing what types of insects visit what types of flowers? Again, the discussion should be more concise and point only to the hypothesis stated. In addition, no mention about urban green management was done.

- "native and non-native plants differ in several" … It is too vague, state which indices differ
- "the number of species and interactions that persist across several periods" … but this is unlikely since pollinator species that persist throughout the year or flowering period are few and often not "strong" pollinators. Solitary wild bees have flight periods of around 2-3 months at most. Only eusocial species cover the entire season with their workers. This applies only if the analysis were done with pollinators identified at species-level, but I don't think so.
- "Urban parks represent artificial assemblages of ornamental plants" … but this is not true, or at least not always true. There are a lot of native plants in urban parks as well.
