# Supplementary material for: Non-native plant integration into plant-insect pollinator networks in urban parks
Source: PLoS One. 2026 Jul 14;21(7):e0353207. doi: 10.1371/journal.pone.0353207 (PMC13367714; doi:10.1371/journal.pone.0353207)
Supplement: Attachment — Submitted filename: Response to Reviewers.docx [file pone.0353207.s019.docx]

18^th^ March, 2026

Dear Prof. Vicente Martínez López,

We are pleased to submit the revised version of our manuscript (PONE-D-25-67039) now entitled “Non-native plant integration into plant-insect pollinator networks in urban parks” for your consideration as a Research Article in *PLOS ONE*.

We thank the associate editor and the two referees for their general positive and insightful comments. We have revised the manuscript in light of these comments, and we believe these changes have strengthened the new version of the manuscript.

Some of the requests made are presented in the recently published paper titled “Year-round pollinator visitation of ornamental plants in Mediterranean urban parks” by Trillo *et al.* 2026. Trillo *et al.* 2026 and our manuscript use the same dataset but do not overlap in their aims and content. Trillo *et al.* 2026 focus on the identity of the plants and pollinators and management practices in urban green areas, while our focus is on the structure of plant-pollinator networks. Therefore, some of the questions made by referees related to the natural history of the species and sampling methods have not been extensively described in our manuscript but referred to Trillo *et al.* 2026.

We provide below responses to the suggestions raised by the associate editor and reviewers’ comments, with a full accounting of the changes.

Thank you for your time and consideration.

On behalf of all co-authors, yours sincerely,

Laura Matas-Granados

Dear Dr. Matas-Granados,

Thank you for submitting your manuscript to PLOS ONE. After careful consideration, we feel that it has merit but does not fully meet PLOS ONE’s publication criteria as it currently stands. Therefore, we invite you to submit a revised version of the manuscript that addresses the points raised during the review process.

Both reviewers acknowledge the interest and relevance of the topic addressed in your manuscript. However, several issues require clarification before the manuscript can be further considered. In the revised version, please include line numbers to facilitate the evaluation process. In particular, some methodological aspects need to be described more clearly. The assumption of independence among pollinator samplings is mentioned but should be explained more explicitly. Additionally, please specify the focal insect group considered in the study (e.g., all pollinators, bees only or bees and hoverflies). Overall, I believe that after a careful revision addressing these points, the manuscript could become suitable for publication.

[Response]: We are grateful for your positive assessment of our work. We are very sorry for the lack of line numbers in the previous version of the manuscript. In this new version, we have included them. We provide below responses to the reviewers’ comments to clarify the sampling design and other methodological details. We also indicate the focal insect groups of study (Hymenoptera, Diptera, Lepidoptera and Coleoptera).

Reviewer #1: Comments to the MS: PONE-D-25-67039

I was given the manuscript entitled “Dynamics of non-native plant integration into plant-pollinator networks in urban parks” The manuscript deals with the role on native and non-native flowering plants in pollination network in Sevilla (Spain). I would point out that without line numbers, the process of reviewing gets incredibly more difficult as I have no means of indicating the point of my comments. I would kindly ask the authors to insert line numbers in the other rounds of revision, thanks.

[Response]: We are very sorry about the omission of line numbers. In this new version, we have inserted them.

General opinion: This study aims to understand the temporal dynamics between plants and pollinators in a city over the course of a year, which is a nice new element supporting the study. However, I believe that it cannot be published in its current form because it is too vague in its identification of plants and pollinating insects (there is never a scientific name or reference). How can one discuss a network without taking into account the entities involved? In addition, although the authors should be commended for the amount of analysis they have done, there are some gaps in the presentation and clarity. I would recommend summarising the information and accompanying it with a few informative graphs and tables. The conclusions are very vague; for example, there are no suggestions for urban green management, no examples of “virtuous plants” or “rejected plants” in these networks. In short, the taxonomic and applicative part is practically absent.

I would suggest the authors to include more recent and relevant literature about their topic, especially given the fact that they have included barely any references to other urban plant-pollinator networks in Europe. https://doi.org/10.1111/1365-2435.13642, https://doi.org/10.1016/j.agee.2021.107560, https://doi.org/10.1556/168.2017.18.1.1, https://doi.org/10.1111/1365-2664.14168, https://doi.org/10.1016/j.ufug.2024.128650

[Response]: We thank the reviewer for these constructive comments on our work.

We need to clarify that the main objective of our work was to explore the differences between non-native and native plants in structuring plant-pollinator networks within an experimental system (i.e. urban green areas) that could simulate a potential highly-invaded natural ecosystem. Our intention was not to give descriptive details of species identities nor to develop recommendations for urban management, but rather to compare the role of plant origin on the architecture of plant-pollinator networks and to discuss their potential implications in the context of biological invasion. Our focus is thus on changes in variables at the community level rather than at the species level. Most of the studies suggested by the reviewer focused on the effects of urbanization gradients, urban habitat characteristics, or management practices without considering plant origin, which is our main aim. We have referred studies considering plant origin in urban green areas (e.g. Zaninotto *et al.* 2023).

The recently published paper Trillo *et al.* 2026 use the same dataset to focus directly on species composition and urban management practices in these Mediterranean urban green areas. This paper and our work do not overlap in main aims and content. Trillo *et al.* 2026 focus on the natural history and phenology of species, while our work focusses on the structure of the networks. Nevertheless, we have revised the manuscript to give more detailed information on species taxa (new Figure S2 with species names), and refer to Trillo *et al.* 2026 whenever required.

There are also other more specific comments, which can be found in the bulleted list below. The authors are free to accept or reject my comments following adequate explanation or justification.

[Response]: Thank you very much. We have included our point-by-point responses hereafter.

Title and Abstract: The title does not convey the main findings of the study. The abstract is ok.

[Response]: We have simplified the title to “Non-native plant integration into plant-insect pollinator networks in urban parks” (lines 1-2),

Introduction: The introduction provides with enough detail to understand the topic of the study. I have only some minor comments.

• When pointing out the hypotheses, I would not separate them from the question, but I would rather continue on the same line. In addition, the questions may also be completely removed to keep only the working hypotheses.

[Response]: We have decided to keep questions to help structuring the headings in methods and results. However, we placed hypothesis on the same line than the question (lines 111-131).

• A key point: in the introduction is not mentioned the focal insect taxa of the study, or all insects? Or all animals?

[Response]: We have also included in the introduction the focal insect taxa (lines 107-109):

“We focus on Hymenoptera, Diptera, Lepidoptera and Coleoptera, which are the main pollinators visiting flowers in Mediterranean urban green areas (Silva *et al.* 2023).”

Methods: The sampling method part needs to be clarified because all the information are randomly placed in the text (or missing) in my opinion. Clearly separate a section for the plant surveys, and a section for pollinator counting. How were the pollinators identified and counted? At which taxonomic resolution? How the authors dealt with the possibility of pseudo-replication in pollinators?

[Response]: A full description of the sampling method is now published in Trillo *et al.* 2026. We respond to your questions below. However, in this new version we did not explain the sampling protocol in detail but summarize it as follows (lines 134-149):

“The study was conducted between October 2021 and September 2022 in 15 urban green areas (i.e., parks) within the city of Seville, in southern Spain. In this city, owing to gardening and consistently warm temperatures throughout the year, floral resources are available nearly year-round. In each park, flowering ornamental woody plant species (both native and non-native) were sampled once a month. The plant and insect pollinator sampling protocol are described in Trillo *et al.* 2026. In sum, in each park flowering plant species which attracted pollinators were sampled across the entire area of the park. Each month, for each plant species, pollinators were sampled in two censuses located in two randomly selected plants along the park. In a census, a 10-minute pollinator observation over a 1 m^2^ area of the flowering plant species was conducted. Within a census any Hymenoptera, Diptera (>3 mm size), Lepidoptera and Coleoptera (>3 mm size) that contacted any reproductive part of a flower was identified and counted, avoiding double counting. Most specimens were identified visually in the field to the lowest possible taxonomic level, except 7.5% of them that were captured for identification in the laboratory with the help of an expert (Francisco P. Molina). Total sampling was 258 hours across 64 days (S1 Table).”

• 20-m wide or long? Transects are usually 2m wide and 50-100m long

[Response]: We sampled plants along a representative 20 m-wide path, which covered the entire area of the park. We have rewritten this part of the methods and refer to Trillo *et al.* 2026 when needed.

• “for each plant” … species I would guess no?

[Response]: From each species, we sampled pollinators on several plants. It is thus correct to say that during each census, we conducted a 10-min pollinator count per plant.

• How can the authors avoid double counting if they just visually counted the pollinators? How can you distinguish between two individuals of the same species from the same individual? All this part is very confusing to me.

[Response]: As in most studies based on visual observations of flower visitors, completely avoiding the possibility of recounting the same individual is almost impossible. However, during the surveys, we attempted to minimize double counting by tracking individual movements within the same focal plant. When a pollinator of a given species was observed moving between nearby flowers and returning to the same plant, it was not counted again. It is possible that in some cases the same individual may have been recorded more than once in different focal plants or species. However, each observation period lasted only 10 minutes and multiple flowering plants were available in the surroundings, which reduces the likelihood that the same individual repeatedly visited the same focal plant within the sampling period. Further, pollinators usually avoid recently visited flowers (Jones *et al.* 1998). In any case, any potential overestimation of visitation rates would affect all plants and parks in a similar way, meaning that relative comparisons among parks remain robust.

• “We sampled 62 plant taxa” … this is a result, not a pre-requisite (method) of the study.

[Response]: We placed the number of taxa in methods because now we refer to the sampling and identification of species described in Trillo *et al.* 2026.

• If those two parks were rather poor in plant taxa, why just not excluding them? The number of parks would still be high enough for robust analysis.

[Response]: These two parks did not receive pollinator visits during two of the four periods considered. However, we chose to retain them in the analyses in order to preserve the full range of variation in plant-pollinator interactions and to avoid selectively excluding parks with low pollination activity in some months of the year. Having said that, if the reviewer and the editor consider their exclusion necessary to ensure the robustness of our results, we are open to repeat the analyses without these two parks and report the results accordingly.

• Chao1 index: How were the pollinators identified and counted? At which taxonomic resolution? How the authors dealt with the possibility of pseudo-replication in pollinators? Presence (0) – absence (1). Because how can you record abundance if you just visually monitored the insects. Again, are you dealing with insects or all animals?

[Response]: The pollinators were identified at the species level by visual observation in the field by Trillo *et al.* 2026 or in the laboratory by an expert (Francisco P. Molina) when identification was not possible in the field.

Abundance was estimated in a 1 m^2^ area with the highest flower density for 10 minutes. See our response above related to avoiding double counting in pollinators.

• Appendix S2: No, I would suggest putting the definition of the metrics here in the main text, they are essential to the readers to understand the study.

[Response]: In this new version, we have included the description of the species-level network parameters in the main text (lines 173-196).

• In building GLMMs, have the authors checked for collinearity? Because I would think that in May-August (period) one should also have higher flower abundance; but these two predictors have been included seemingly blindly.

[Response]: We agree about testing the potential collinearity between predictors. We assessed collinearity among predictors using generalized variance inflation factors (GVIFs) for models with some categorical predictors (Fox & Monette 1992). All values for all models were below 1.6, indicating no problematic collinearity among predictors. Therefore, including period and floral availability in the models does not affect the interpretation of the best model. We included this aspect in the methodology (lines 223-225):

“We assessed potential collinearity among predictors for models using generalized variance inflation factors (GVIFs) (Fox & Monette 1992). All values for all models were below 1.6, indicating no problematic collinearity among predictors.”

• I understand the usage of AIC, but this comes with a non-negligible problem: different metrics on the same network are analysed in different ways, which can lead to misleading interpretations. Why do not choose a single model that answers all the questions posed by the authors and use it straightforward? This would also simplify the reading and understanding of the results.

[Response]: Thank you for this consideration. Selecting models using AIC for each species-level network parameter is the most statistically robust approach, since each species-level network parameter explain a different characteristic of the network structure. Therefore, each parameter may have related to different predictors, and forcing a single model for all metrics could lead to overfitting or the inclusion of irrelevant predictor for some parameters. Nevertheless, we understand the complexity in the results considering a different best model for each parameter and we have made an effort reducing non-significant results in new Figure 1.

Results: The presentation of the results is insufficient. There are no tables... and the text does not even contain the minimum parameters needed to understand the statistical comparisons/models. Everything needs to be formalised. In this form, the results are barely understandable. The more if you consider that the authors have done a lot of analysis, but in this form, everything is very confusing. I suggest the authors reducing and formalising this section, making it more precise. Now there are a lot of statements, supported by supplementary tables that are not immediately readable. The results should be concise, accompanied by a few extremely significant and informative tables and figures and, given that the authors have carried out numerous analyses, divided by “hypothesis” or “question”.

[Response]: We have clarified the presentation of the results by: (i) modifying section labels to match those in methods and to relate them to the research questions in the introduction, (ii) removing non-significant results and adding tables related to main results for questions 1 and 4. We hope these changes make results more understandable.

• Fig.S2 is almost incomprehensible. First, the four periods are in square 2x2 (which I figured out after a while), second there are no names about the interacting species. At the moment, this figure does not provide relevant information and can therefore be removed or refined.

[Response]: Thank you for the comment. Our intention with this graph was to offer a visual representation of how plant-pollinator interactions change over periods depending on plant origin. We understand the overwhelming information representing all parks separately. For visual clarity, we changed Fig. S2 to four panels, one for each period, pulling plant-pollinator interactions of all parks together. We have also included species names.

• Fig.1 please add statistically significant comparisons to the boxplots for all the comparisons OR delete plots showing non-significant comparisons. In addition, where are the tables with the selected models? They were not even mentioned in the text the summary statistics of the models

[Response]: In Fig. 1 we have removed results with non-significant differences. We have also moved subpanels *f* and *h* as a supplementary figure (Figure S5). We have kept previous Table S3 (now Table S4) with the comparison of models by their AIC and the selection of the best model for each parameter in Supplementary Information. We have moved previous Table S4 to the main text (new Table 1).

• Table S4. This table should be placed in the text. Why there is never Period 3? In addition, I think here there is a problem of interpretation. When interactions are statistically significant, they should be discussed as the sole significant terms, and not together with their non-interacting factors. You must interpret the interaction first. The main effects become less relevant or misleading because the effect of that variable isn't consistent (as suggested by the significant interaction).

[Response]: We have moved previous Table S4 to the main text (new Table 1). Period 3 was used as the reference level in the model. Therefore, its mean value is represented by the intercept, and the coefficients for Period 1, 2 and 4 indicate differences relative to Period 3 (same with the level “Native” in the cases where the predictor “Origin” was included in the model). We have clarified it in the table caption.

We agree that when a statistically significant interaction exists, it should be interpreted before and instead of main effects. We have two cases in which an interaction between two predictors appears as significant: (i) for specialization (where the significant interaction was between period and origin) and (ii) for *c* parameter (where the significant interaction was between floral length and floral availability). In (i) the interaction was between two categorical predictors and then we compared the two levels of plant origin within each level of period. We have shown in new Figure 1 the significant differences (only within period 4). In (ii) we have shown the interaction between the two continuous variables floral length and flora availability visually in new Figure S5.

• Fig.3 is almost incomprehensible because it does not convey clear information but rather a chaotic representation of the networks though the four seasons. In addition, again, without names it is even less informative.

[Response]: We thank the reviewer for this comment. The aim of Fig. 3 was to illustrate the seasonal dynamics of plant–pollinator interactions across periods within each park, with particular emphasis on the origin (native vs. non-native) of the plant species involved in each interaction. The figure was intended to provide a visual representation of interaction turnover across seasons. As shown in the figure, most interactions occur within a single period, while fewer interactions persist across two, three, or four periods. Importantly, no consistent seasonal pattern was detected in relation to plant origin, a result that was formally tested using GLMMs with beta diversity interaction (new Table 2). In the figure, each circle (or triangle) represents an interaction plant-pollinator so labelling each circle with plant-pollinator names would be the graph much more incompressible and unclear.

However, we acknowledge that including 15 parks results in a visually dense and less immediately interpretable figure. To improve clarity in the main text, we have moved Fig. 3 to the Supplementary Information and improved its layout (new Figure S7). We have instead moved the previous Table S6 to the main text (new Table 2), as it more directly summarizes the key results related to the fourth question: “How do non-native plant species maintain interaction turnover throughout the year?”.

We believe the network visualization still provides valuable complementary information and therefore we have retained it in the Supplementary Material. Nevertheless, we are open to further adjustments if the reviewer and editor consider additional changes necessary.

Discussion: I believe that, in light of what I have read, the entire discussion and study have a major flaw. The authors did not consider the type of plant (the species are never mentioned) or its floral morphology, nor did they consider the type of insect that visited it. How can one generalise without knowing what types of insects visit what types of flowers? Again, the discussion should be more concise and point only to the hypothesis stated. In addition, no mention about urban green management was done.

[Response]: Thank you for the comment. As mentioned before, the objective of our study was not to generalize about which insect taxa visit particular plant taxa or floral morphological traits, nor offer urban green management help, but to evaluate how plant origin (native vs. non-native) influences on plant-pollinator network structure through time, and discuss these results in the context of biological invasion. Our focus is thus on changes in variables at the community level rather than at the species level. Species level features were analyzed in a previous publication (Trillo *et al.* 2026), where preferences and diversity of insect groups and taxa was explicitly examined.

We have double-checked that each subsection responds to the hypothesis stated.

• “native and non-native plants differ in several” … It is too vague, state which indices differ

[Response]: We have changed it accordingly (lines 386-388):

“native and non-native plants differ in species strength, specialization in the last period of the year, within module degree and among modules connectivity”

• “the number of species and interactions that persist across several periods” … but this is unlikely since pollinator species that persist throughout the year or flowering period are few and often not “strong” pollinators. Solitary wild bees have flight periods of around 2-3 months at most. Only eusocial species cover the entire season with their workers. This applies only if the analysis were done with pollinators identified at species-level, but I don’t think so.

[Response]: Our dataset includes a broad range of pollinator taxa. Many were recorded across multiple months. In Table A2 of Supplementary material of Trillo *et al.* 2026 there is a list of pollinator taxa recorded and their phenology. At least 25 taxa were observed during more than 4-5 months, some were present for most of the year, and many of them were not eusocial species. Although many species have restricted activity periods, a subset of pollinators in our study system does persist across several sampling periods and can contribute to connecting temporal networks. Nevertheless, the number of such species and interactions is relatively small, which is consistent with our observation that only a few interactions persisted through time. Regarding taxonomic resolution, as said before, pollinators were identified to the lowest possible taxonomic level (species when possible, otherwise morphospecies).

• “Urban parks represent artificial assemblages of ornamental plants” … but this is not true, or at least not always true. There are a lot of native plants in urban parks as well.

[Response]: Thank you for the comment. Our intention was not to imply that “ornamental” is synonymous of “non-native”. We focused specifically on ornamental woody plant species, which included both native and non-native taxa. Thus, “ornamental” refers to their use and planting context, not to their biogeographic origin. We have revised the manuscript to clarify that urban parks host a mixture of native and non-native ornamental woody species, and that our comparisons explicitly consider plant origin (native vs. non-native) within this ornamental assemblage (lines 135-138):

“In this city, owing to gardening and consistently warm temperatures throughout the year, floral resources are available nearly year-round. In each park, flowering ornamental woody plant species (both native and non-native) were sampled once a month.”

Reviewer #2: In this study, the authors investigate the dynamics of non-native plant integration into plant–pollinator interaction networks in urban parks of a Mediterranean city over the course of one year. They compare native and non-native plants in terms of their structural roles within the network and their contribution to temporal connectivity and interaction turnover. The authors show that native and non-native plants play distinct structural roles while contributing similarly to the temporal connectivity of the network.

At a time when biological invasions are becoming increasingly frequent and may alter interactions between native plants and pollinators, this study is highly relevant. Overall, I found the manuscript to be well structured, timely, and interesting. However, I have a few minor concerns that I would like to share with the authors to help improve the clarity and robustness of the manuscript. Finally, I congratulate the authors on their effort and hope that my comments will be useful.

[Response]: Thank you very much for your positive comments. We address your concerns below.

Minnor comments

Methods

Parks were used as a random factor in the analyses, but it is not clear how this variable varies. Could you better explain this in the Methods section?

[Response]: We included park as random factor to account for species composition differences among parks. We have added in Methods (lines 202-205):

“Parks also have different sizes (mean = 0.17 km2, range = 0.003–0.511 km2), and are located at varying distances from the city edge to the center (mean = 1.5 km, range = 0.0–3.4 km).”

We have also included a supplementary table with the number of plant and pollinator taxa, and interactions sampled by park (new Table S1).

Were ruderal/spontaneous plants sampled? Please clarify this point and provide a clear definition of what was considered an ornamental plant in the study.

[Response]: As mentioned in the introduction and methods we sampled woody plants, most of them planted.

At which taxonomic level were plants and pollinators identified (e.g., species, genus)? Please clarify this information.

[Response]: Plants were identified at the species level when possible. However, 17 out of 62 plant taxa were grouped by genus because there was a mix of species and hybrids with cryptic floral traits. With regard to pollinators, 50 out of 173 pollinator taxa (29%) could not be identified to species and they were included as morphospecies. We have included this information in the main text (lines 151-156):

“Overall, the plant-pollinator analyses were based on 62 plant taxa (44 non-native and 16 native). Although our aim was to focus at the plant species resolution level, we grouped 17 plant taxa by genus because there was a mix of species and hybrids with cryptic floral traits, which we assumed were equally attractive to pollinators (Trillo *et al.* 2026). These plants were visited by 8422 pollinators belonging to 173 pollinator taxa (99 Hymenoptera, 57 Diptera, 12 Lepidoptera, 5 Coleoptera), 71% identified at the species level.”

Did you calculate sampling effort for each park? I suggest estimating and reporting this information, possibly as a table in the Supplementary Material.

[Response]: We have added a new supplementary table (new Table S1) with sampling effort by park:

Table S1. Sampling effort, total number of plant and pollinator taxa and interactions by park. Time is expressed in minutes.

| Park | Total sampling time | Time census per period | | | | Mean time census per plant species | Nº plants | Nº pollinators | Nº interactions |
| --- | --- | --- | --- | --- | --- | --- | --- | --- | --- |
|  |  | 1 | 2 | 3 | 4 |  |  |  |  |
| Alamillo | 760 | 290 | 160 | 130 | 180 | 54 | 14 | 49 | 98 |
| Álvaro Diamantino Vellisco | 220 | 40 | 120 | 60 | 0 | 37 | 6 | 20 | 32 |
| Amate | 1400 | 470 | 540 | 210 | 180 | 50 | 28 | 57 | 211 |
| Bermejales | 580 | 190 | 140 | 70 | 180 | 58 | 10 | 51 | 102 |
| José Celestino Mutis | 1240 | 430 | 440 | 190 | 180 | 44 | 28 | 40 | 155 |
| Federico García Lorca | 620 | 100 | 200 | 220 | 100 | 69 | 10 | 37 | 79 |
| Infanta Elena | 610 | 290 | 160 | 70 | 90 | 41 | 17 | 50 | 103 |
| Jardines de la Buhaira | 530 | 210 | 190 | 60 | 70 | 48 | 11 | 37 | 83 |
| Jardines del Guadalquivir | 1060 | 380 | 400 | 170 | 110 | 39 | 27 | 64 | 192 |
| Jardines del Valle | 890 | 300 | 270 | 200 | 120 | 56 | 16 | 44 | 129 |
| Jose María de los Santos | 620 | 220 | 150 | 130 | 120 | 52 | 12 | 45 | 108 |
| María Luisa | 1130 | 320 | 420 | 260 | 130 | 43 | 26 | 45 | 177 |
| Don Miguel Mañara | 130 | 60 | 30 | 40 | 0 | 33 | 4 | 13 | 18 |
| Parque de los Príncipes | 1320 | 330 | 560 | 310 | 120 | 53 | 25 | 52 | 190 |
| Tamarguillo | 950 | 280 | 380 | 170 | 120 | 50 | 19 | 66 | 154 |

Did you calculate the sampling completeness of the interaction networks? Although network size was included as a random factor, it would be informative to show how well sampled these networks were.

[Response]: We calculated sampling completeness of interactions by park and period (new Table S2).

Why did you use a binary interaction matrix to calculate centrality metrics? Given that you appear to have interaction frequency data, it would be interesting to use quantitative matrices and explore weighted metrics (e.g., weighted closeness and weighted betweenness). The same point applies to beta diversity analyses. Why were quantitative interaction matrices not used to calculate interaction’s beta diversity?

[Response]: Thank you for the insight. These two analyses are related to our third and fourth question in which, in summary, we wanted to know the role of non-native plant species connecting temporal networks and maintaining interaction turnover. Our objectives were more centered on changes in the identity of interactions rather than on variation in interaction strength. For this reason, we use binary matrices instead of quantitative matrices.

Results

Please indicate the total number of pollinator species recorded in the study. In addition, a brief description comparing plants, pollinators, and interaction diversity among the parks would help to better contextualize the results.

[Response]: This information is now published in Trillo *et al.* (2026) and we summarized the main results in Methods to describe the dataset we used to build the plant-pollination networks. Notice that both studies do not overlap in aims. The information on number of plant and pollinator taxa and interactions per park can be found in new Table S1.

Discussion

The finding that non-native plants tend to overlap less in flowering with other non-native plants is very interesting and could be highlighted more explicitly in the Discussion.

Please provide more details on how the relationships among native–native, non-native–non-native, and native–non-native plant interactions relate to competition and facilitation processes.

[Response]: We have added further details related to these issues in the Discussion (lines 442-455):

“Thus, the frequent co-flowering of native–non-native pairs, alongside the relative scarcity of synchronous non-native–non-native pairs, may play an important role in structuring urban pollination networks. To disentangle the ecological processes underlying these patterns, it is necessary to track pollen fluxes between native and non-native species and the consequences for plant reproduction (Alarcón 2010). Such analyses could clarify the balance of facilitative and competitive interactions shaping these networks (Emer *et al.* 2015; Lopezaraiza–Mikel *et al.* 2007). A global analysis between neighbour plant species found an overall significantly negative effect of non-native plants on visitation rates of pollinators and reproduction of co-flowering native plants, being most detrimental when the non-native and the native species had similar flower symmetry or colour. In contrast, the effect of native plants on visitation and reproduction of neighbour native plant was non-significant (Morales & Traveset 2009). These results indicate that pollinator and pollination competition between non-native and native plants might be common.”

Supplementary Material

The title of Table S1 should be placed above the table, not below it.

[Response]: We have changed it.

For the model including within-degree z, the AIC of the model with flowering length as the only predictor and park as a random factor is lower than the others (AIC = 540.8312). If this interpretation is correct, this should be considered the best model, and the corresponding results (Table S3, Table S4, and related text) may need to be adjusted. Why did you not use the delta AIC?

[Response]: The model including flowering length as the only fixed predictor showed a slightly lower AIC (540.8312). However, the difference in AIC between this model and the selected model was < 2 and therefore, they are considered to have equivalent empirical support. Therefore, there is no strong statistical evidence to clearly prefer one model over the other (Burnham & Anderson 2004). In this unique case, we decided to show the results of the model including plant origin which is related to our question (comparison network structure considering plant origin).

While AIC values were already presented in new Table S4 (from which ΔAIC can be directly calculated), we agree that reporting ΔAIC explicitly improves clarity. We have therefore added ΔAIC values to the table and clarified in the Methods section that models with ΔAIC < 2 were considered to have comparable support (lines 228-233):

“Models were compared using ΔAIC as the difference between the AIC value of the model and the lowest AIC observed among the candidate set (ΔAIC = AICᵢ − AIC_min_). Models with ΔAIC > 2 indicated that the worst model had no support and could be omitted (Burnham & Anderson 2004). Models with ΔAIC ≤ 2 were considering strongly supported. When more than one model had ΔAIC ≤ 2, we decided showing the results of the model that included plant origin.”

Figures

Please standardize terminology across all figures (e.g., “Non-native” vs. “Nonnative”).

In addition, the different levels of categorical variables should start with capital letters (e.g., “Mix”, “Native”, “Non-native”).

[Response]: We have corrected both aspects in the new version of the manuscript.

Data availability

I strongly suggest that the authors provide a list of plant species, pollinator species, and recorded interactions. I was not able to access the Zenodo link provided, so I could not verify whether these data are already available.

[Response]: List of plant and pollinator species are shown in the supplementary material of Trillo *et al.* 2026.

In the Zenodo link we have made available all plant-pollinator interactions recorded by census and park as well as R script to reproduce analyses and results.

**References**

Alarcón, R. (2010). Congruence between visitation and pollen-transport networks in a California plant–pollinator community. *Oikos*, 119, 35–44.

Burnham, K.P. & Anderson, D.R. (Eds.). (2004). *Model Selection and Multimodel Inference*. Springer, New York, NY.

Emer, C., Vaughan, I.P., Hiscock, S. & Memmott, J. (2015). The Impact of the Invasive Alien Plant, Impatiens glandulifera, on Pollen Transfer Networks. *PLOS ONE*, 10, e0143532.

Fox, J. & Monette, G. (1992). Generalized Collinearity Diagnostics. *J. Am. Stat. Assoc.*, 87, 178–183.

Jones, K.N., Reithel, J.S. & Irwin, R.E. (1998). A trade-off between the frequency and duration of bumblebee visits to flowers. *Oecologia*, 117, 161–168.

Lopezaraiza–Mikel, M.E., Hayes, R.B., Whalley, M.R. & Memmott, J. (2007). The impact of an alien plant on a native plant–pollinator network: an experimental approach. *Ecol. Lett.*, 10, 539–550.

Morales, C.L. & Traveset, A. (2009). A meta-analysis of impacts of alien vs. native plants on pollinator visitation and reproductive success of co-flowering native plants. *Ecol. Lett.*, 12, 716–728.

Silva, V.H.D., Gomes, I.N., Cardoso, J.C.F., Bosenbecker, C., Silva, J.L.S., Cruz-Neto, O., *et al.* (2023). Diverse urban pollinators and where to find them. *Biol. Conserv.*, 281, 110036.

Trillo, A., Ragel-Celdrán, D., Molina, F.P. & Vilà, M. (n.d.). Year-round pollinator visitation of ornamental plants in Mediterranean urban parks. *Insect Conserv. Divers.*, n/a.

Zaninotto, V., Thebault, E. & Dajoz, I. (2023). Native and exotic plants play different roles in urban pollination networks across seasons. *Oecologia*, 201, 525–536.
