# Supplementary material for: Non-native plant integration into plant-insect pollinator networks in urban parks
Source: PLoS One. 2026 Jul 14;21(7):e0353207. doi: 10.1371/journal.pone.0353207 (PMC13367714; doi:10.1371/journal.pone.0353207)
Supplement: Attachment — Submitted filename: Comments_PONE-D-25-67039_R1.docx [file pone.0353207.s018.docx]

**Comments to the MS: PONE-D-25-67039_R1**

I was given the revised version of the manuscript now entitled “*Non-native plant integration into plant-insect pollinator networks in urban parks*”

Overall, the manuscript has been improved and I think, if other reviewers and editors agree, merits publication. I have only few minor comments that the author can easily resolve without a rebuttal letter.

The introduction was already well presented and therefore didn’t need any further comments.

The methods are now cleared and better divided into subsections. While I do not like referencing to other papers in the methods (because I would like to read it in the paper I selected), I understand the methods are those from Trillo. I don’t like the choice, but I respect it.

- L212: Km ^2^

The results are now clearer with these subheadings.

- In table 1, Among-module connectivity, P = 0.055 should not be boldfaced, it is not statistically significant.

The discussion should be adjusted. First, I would not separate the discussion into subsections, but I would rather make a cohesive, concise (!!!), and informative section. I recommend the authors to short the discussion to really highlight what they have found. Instead use paragraphs to separate arguments. Furthermore, I must unfortunately accept that I will not see any plant names listed, nor any mention of their potential positive or negative role within the network. I find it a major shortcoming that no plants are identified as keystone species for urban networks. This study fails to offer any practical applications, despite its great potential to do so. Instead, however, it engages in extensive speculation on purely theoretical network concepts, which I find of little interest.

- L385: six rows for a single sentence are way too much, please rephrase.
