# Supplementary material for: Non-native plant integration into plant-insect pollinator networks in urban parks
Source: PLoS One. 2026 Jul 14;21(7):e0353207. doi: 10.1371/journal.pone.0353207 (PMC13367714; doi:10.1371/journal.pone.0353207)
Supplement: Attachment — Submitted filename: Response_to_Reviewers_auresp_2.docx [file pone.0353207.s020.docx]

22^nd^ May 2026

Dear Prof. Vicente Martínez López,

Thank you for your assessment and positive feedback from both reviewers regarding the improvements made in the revised version.

We have carefully considered all remaining comments and concerns raised by the reviewers. In response, we have further strengthened the Discussion section, as suggested by Reviewer 1, clarified the methodology and added new tables as recommended by Reviewer 2. Detailed responses to each comment, together with the corresponding changes made in the manuscript, are provided in the revised response document.

We hope that the revised version will now be suitable for publication in Plos ONE.

Laura Matas-Granados

Dear Dr. Matas-Granados,

Both reviewers acknowledge the improvement of the manuscript after revision. However, they still identify several issues that should be addressed before the manuscript can be considered for publication. In particular, Reviewer 1 suggests further improvements to the Discussion section, while Reviewer 2 highlights some aspects of the methodology and results that require clarification. Please carefully address these remaining concerns in a revised version of the manuscript, and I will be pleased to reconsider it for publication in PLOS ONE.

[Response]: Thank you for the assessment and the positive feedback. Find below our responses to reviewers’ comments.

Reviewer #1: Comments to the MS: PONE-D-25-67039_R1

I was given the revised version of the manuscript now entitled “Non-native plant integration into plant-insect pollinator networks in urban parks”
Overall, the manuscript has been improved and I think, if other reviewers and editors agree, merits publication. I have only few minor comments that the author can easily resolve without a rebuttal letter.

The introduction was already well presented and therefore didn’t need any further comments.

The methods are now cleared and better divided into subsections. While I do not like referencing to other papers in the methods (because I would like to read it in the paper I selected), I understand the methods are those from Trillo. I don’t like the choice, but I respect it.

[Response]: Thank you for the assessment. To improve clarity and make the methodology easier to follow for readers; we have now transcribed verbatim the sampling design methodology originally described by Trillo *et al.* 2026 into the Supplementary Material (S2 Appendix). We hope that this addition will not be considered plagiarism. We are open to remove it if you consider it.

We added in the text:

Lines 152-154: “For further detailed information regarding the sampling design, please refer to Trillo *et al.* 2026 (transcribed verbatim in S2 Appendix)”.

• L212: Km 2

[Response]: We have changed it accordingly.

The results are now clearer with these subheadings.

• In table 1, Among-module connectivity, P = 0.055 should not be boldfaced, it is not statistically significant.

[Response]: We have not boldfaced marginal significant terms; instead, we indicated them with a “+” symbol.

The discussion should be adjusted. First, I would not separate the discussion into subsections, but I would rather make a cohesive, concise (!!!), and informative section. I recommend the authors to short the discussion to really highlight what they have found. Instead use paragraphs to separate arguments. Furthermore, I must unfortunately accept that I will not see any plant names listed, nor any mention of their potential positive or negative role within the network. I find it a major shortcoming that no plants are identified as keystone species for urban networks. This study fails to offer any practical applications, despite its great potential to do so. Instead, however, it engages in extensive speculation on purely theoretical network concepts, which I find of little interest.

[Response]: Following your recommendation, we have revised and shortened the Discussion by 27% to improve its clarity and conciseness. We removed the previous subsection structure and reduced extensive theoretical considerations on network concepts in order to focus more directly on the main findings of the study.

In addition, we incorporated specific examples of plant species and key plant–pollinator interactions identified in our results to better support our interpretations and increase the practical relevance of the study:

Lines 399-401: “However, contrary to our hypothesis, non-native plants, such as *Pyracantha coccinea* or *Lantana* spp., displayed greater specialization than native plants during resource-scarce periods.”

Lines 404-407: “In contrast, native plants exhibited greater species strength (e.g., *Salvia rosmarinus*), indicating that a wide range of pollinators depend on them when available, highlighting their role in sustaining network stability.”

Lines 408-410: “As expected, non-native plants showed higher among-module connectivity (e.g., *Agapanthus africanus*), whereas natives exhibited higher within-module degree (e.g., *Salvia rosmarinus*).”

Lines 420-423: “However, native–non-native pairs forming modules tended to co-flower more frequently than non-native–non-native pairs. This is illustrated by examples such as the pair *Lavandula* spp. (native) and *Lantana* spp. (non-native) which co-flowered during three of the four periods.”

Lines 450-454: “This high turnover aligns with the finding that only a few interactions connected temporal networks over the course of the year. This will be the case for some interactions between *Apis mellifera* and *Xylocopa violacea* with both native (e.g., *Lavandula* spp.) and non-native plants (e.g., *Duranta erecta)*, underscoring the transient nature of most plant–pollinator interactions.

• L385: six rows for a single sentence are way too much, please rephrase.

[Response]: We have simplified it accordingly:

Lines 392-396: “Our study examining year-round plant-pollination interactions in urban green areas indicate that native and non-native plants differ on how they integrate into the plant-pollinator network and in their modules. However, native and non-native plants have similar capacities as temporal network connectors, and on their contribution to interaction turnover.”

Reviewer #2: The authors have done an excellent job in improving this new version of the manuscript. The study is now much more consistent with the questions and hypotheses being tested, and I would like to congratulate them on these improvements.

However, I still have a few concerns. As the authors note, this study and Trillo et al. (2026) used the same dataset. Although the main objective of the present work is not directly focused on species identity or management practices in urban areas, these aspects are highly relevant when working with interaction networks in urban green species. In this context, the inclusion of species names in the bipartite networks is an important and welcome improvement.

In addition, I strongly suggest the inclusion of two new supplementary tables: one presenting the plant species along with relevant traits (e.g., origin), and another with information on the pollinators. I also encourage the authors to make the interaction data publicly available (e.g., via Zenodo).

[Response]: Thank you for the assessment. We have now included in the supplementary information a table with the information of plant species sampled (new S2 Table) and another table with pollinator species sampled (new S3 Table). We have also included as publicly available a table with all the plant-pollinator interactions recorded (“Table_interactions.csv” in <https://doi.org/10.20350/digitalCSIC/18370> in DIGITAL CSIC and <https://doi.org/10.5281/zenodo.20305055> in Zenodo).

The study relies on visual observations, and as nicely pointed out by Reviewer 1, it is still unclear how the models address the issue of pseudoreplication. If this issue is not accounted for in the analyses, I suggest that it be explicitly acknowledged as a limitation of the study.

[Response]: We avoided counting the same individual visiting different flowers during a census. However, as we mentioned in our previous response, we acknowledge that, as in most studies based on visual observations of flower visitors, it is not possible to completely avoid pseudoreplication, because the same individual pollinator may be recorded more than once if it departs away from the sampling area and comes back during the sampling period. We have now included this limitation of the study.

Lines 144-149: “Within a census any Hymenoptera, Diptera (>3 mm size), Lepidoptera and Coleoptera (>3 mm size) that contacted any reproductive part of a flower was identified and counted. We avoided double counting the same individual within the 1 m^2^ area sampling area. However, we acknowledge that pseudoreplication could not be entirely excluded if, for instance, the individual leaves the sampling area and comes back.”

Regarding the methods, it would be helpful to clarify whether the sampling design was based on a transect or a plot. If it was a transect, as it seems, please specify its length, given that its width is reported as 20 m.

[Response]: Surveys were conducted in a representative sampling plot of the park. This plot was 20 m-wide with a length that covered the entire extent of each park. Therefore, the length of the plot varied depending on park size, because the objective was to comprehensively sample all flowering woody plant species within each park. We have included in methods:

Lines 137-139: “In each park, we selected a representative 20 m-wide plot with a length that covered the entire extent of each park. In each plot, flowering ornamental woody plant species (both native and non-native) were sampled once a month.”

Line 337: Please check the results, as the nested contribution does not appear to decrease with network size.

[Response]: We have changed it accordingly:

Lines 342-345: “Normalized degree, species strength, and contribution to nestedness increased with flower availability, whereas normalized degree and species strength decreased, and contribution to nestedness increased, with larger network size (Table 1; S4 Fig).”

Table S1: I suggest including the standard deviation in the column “Mean time census per plant species” to better represent the variation.

[Response]: We have added the standard deviation to the mean time census per plant species.

Figure S2: Please clarify in the legend what the colours in the boxes represent for each species, especially for plants. It would be helpful to indicate whether the colours correspond to species origin (native vs. non-native), as this seems to be the case but is not explicitly stated. For pollinators, you may consider colouring the boxes according to the different orders recorded (e.g., Hymenoptera, Diptera, Lepidoptera, and Coleoptera).

[Response]: We have added to the legend the significance of each colours for plant and pollinator taxa. Colours for plant taxa represent their plant origin and now colors for pollinator taxa represent their order.
