# Supplementary material for: Non-native plant integration into plant-insect pollinator networks in urban parks
Source: PLoS One. 2026 Jul 14;21(7):e0353207. doi: 10.1371/journal.pone.0353207 (PMC13367714; doi:10.1371/journal.pone.0353207)
Supplement: Peer review history

# Peer review: Decision Letter 0

**DOI:** 10.1371/journal.pone.0353207.r001

**Authors:** Vicente Martínez López

19 Feb 2026

PONE-D-25-67039

Dynamics of non-native plant integration into plant-pollinator networks in urban parks

PLOS One

Dear Dr. Matas-Granados,

Thank you for submitting your manuscript to PLOS ONE. After careful consideration, we feel that it has merit but does not fully meet PLOS ONE’s publication criteria as it currently stands. Therefore, we invite you to submit a revised version of the manuscript that addresses the points raised during the review process.

Both reviewers acknowledge the interest and relevance of the topic addressed in your manuscript. However, several issues require clarification before the manuscript can be further considered. In the revised version, please include line numbers to facilitate the evaluation process. In particular, some methodological aspects need to be described more clearly. The assumption of independence among pollinator samplings is mentioned but should be explained more explicitly. Additionally, please specify the focal insect group considered in the study (e.g., all pollinators, bees only or bees and hoverflies). Overall, I believe that after a careful revision addressing these points, the manuscript could become suitable for publication.

Please submit your revised manuscript by Apr 05 2026 11:59PM. If you will need more time than this to complete your revisions, please reply to this message or contact the journal office at plosone@plos.org. When you're ready to submit your revision, log on to https://www.editorialmanager.com/pone/ and select the 'Submissions Needing Revision' folder to locate your manuscript file.

Please include the following items when submitting your revised manuscript:

- A letter that responds to each point raised by the academic editor and reviewer(s). You should upload this letter as a separate file labeled 'Response to Reviewers'.
- A marked-up copy of your manuscript that highlights changes made to the original version. You should upload this as a separate file labeled 'Revised Manuscript with Track Changes'.
- An unmarked version of your revised paper without tracked changes. You should upload this as a separate file labeled 'Manuscript'.

If you would like to make changes to your financial disclosure, please include your updated statement in your cover letter. Guidelines for resubmitting your figure files are available below the reviewer comments at the end of this letter.

If applicable, we recommend that you deposit your laboratory protocols in protocols.io to enhance the reproducibility of your results. Protocols.io assigns your protocol its own identifier (DOI) so that it can be cited independently in the future. For instructions see: https://journals.plos.org/plosone/s/submission-guidelines#loc-laboratory-protocols. Additionally, PLOS ONE offers an option for publishing peer-reviewed Lab Protocol articles, which describe protocols hosted on protocols.io. Read more information on sharing protocols at https://plos.org/protocols?utm_medium=editorial-email&utm_source=authorletters&utm_campaign=protocols.

We look forward to receiving your revised manuscript.

Kind regards,

Vicente Martínez López

Academic Editor

PLOS One

Journal Requirements:

When submitting your revision, we need you to address these additional requirements.

1. Please ensure that your manuscript meets PLOS ONE's style requirements, including those for file naming. The PLOS ONE style templates can be found at

https://journals.plos.org/plosone/s/file?id=wjVg/PLOSOne_formatting_sample_main_body.pdf and https://journals.plos.org/plosone/s/file?id=ba62/PLOSOne_formatting_sample_title_authors_affiliations.pdf

2. Please note that PLOS One has specific guidelines on code sharing for submissions in which author-generated code underpins the findings in the manuscript. In these cases, we expect all author-generated code to be made available without restrictions upon publication of the work. Please review our guidelines at https://journals.plos.org/plosone/s/materials-and-software-sharing#loc-sharing-code and ensure that your code is shared in a way that follows best practice and facilitates reproducibility and reuse.

3. Thank you for stating in your Funding Statement:

[This research was supported by the project RADIOPOPO (PID2021-122690OB-I00) funded by MCIN/AEI/10.13039/501100011033/FEDER, UE.].

Please provide an amended statement that declares *all* the funding or sources of support (whether external or internal to your organization) received during this study, as detailed online in our guide for authors at http://journals.plos.org/plosone/s/submit-now.  Please also include the statement “There was no additional external funding received for this study.” in your updated Funding Statement.

Please include your amended Funding Statement within your cover letter. We will change the online submission form on your behalf.

4. When completing the data availability statement of the submission form, you indicated that you will make your data available on acceptance. We strongly recommend all authors decide on a data sharing plan before acceptance, as the process can be lengthy and hold up publication timelines. Please note that, though access restrictions are acceptable now, your entire data will need to be made freely accessible if your manuscript is accepted for publication. This policy applies to all data except where public deposition would breach compliance with the protocol approved by your research ethics board. If you are unable to adhere to our open data policy, please kindly revise your statement to explain your reasoning and we will seek the editor's input on an exemption. Please be assured that, once you have provided your new statement, the assessment of your exemption will not hold up the peer review process.

5. Please note that your Data Availability Statement is currently missing the repository name. If your manuscript is accepted for publication, you will be asked to provide these details on a very short timeline. We therefore suggest that you provide this information now, though we will not hold up the peer review process if you are unable.

If the reviewer comments include a recommendation to cite specific previously published works, please review and evaluate these publications to determine whether they are relevant and should be cited. There is no requirement to cite these works unless the editor has indicated otherwise.

[Note: HTML markup is below. Please do not edit.]

Reviewers' comments:

Reviewer's Responses to Questions

**Comments to the Author**

1. Is the manuscript technically sound, and do the data support the conclusions?

The manuscript must describe a technically sound piece of scientific research with data that supports the conclusions. Experiments must have been conducted rigorously, with appropriate controls, replication, and sample sizes. The conclusions must be drawn appropriately based on the data presented.

Reviewer #1: Partly

Reviewer #2: Yes

**********

2. Has the statistical analysis been performed appropriately and rigorously?

Reviewer #1: Yes

Reviewer #2: Yes

**********

3. Have the authors made all data underlying the findings in their manuscript fully available?

The PLOS Data policy requires authors to make all data underlying the findings described in their manuscript fully available without restriction, with rare exception (please refer to the Data Availability Statement in the manuscript PDF file). The data should be provided as part of the manuscript or its supporting information, or deposited to a public repository. For example, in addition to summary statistics, the data points behind means, medians and variance measures should be available. If there are restrictions on publicly sharing data—e.g. participant privacy or use of data from a third party—those must be specified.

Reviewer #1: No

Reviewer #2: Yes

**********

4. Is the manuscript presented in an intelligible fashion and written in standard English?

PLOS ONE does not copyedit accepted manuscripts, so the language in submitted articles must be clear, correct, and unambiguous. Any typographical or grammatical errors should be corrected at revision, so please note any specific errors here.

Reviewer #1: Yes

Reviewer #2: Yes

**********

5. Review Comments to the Author

Please use the space provided to explain your answers to the questions above. You may also include additional comments for the author, including concerns about dual publication, research ethics, or publication ethics. (Please upload your review as an attachment if it exceeds 20,000 characters)

Reviewer #1: Comments to the MS: PONE-D-25-67039

I was given the manuscript entitled “Dynamics of non-native plant integration into plant-pollinator networks in urban parks” The manuscript deals with the role on native and non-native flowering plants in pollination network in Sevilla (Spain). I would point out that without line numbers, the process of reviewing gets incredibly more difficult as I have no means of indicating the point of my comments. I would kindly ask the authors to insert line numbers in the other rounds of revision, thanks.

General opinion: This study aims to understand the temporal dynamics between plants and pollinators in a city over the course of a year, which is a nice new element supporting the study. However, I believe that it cannot be published in its current form because it is too vague in its identification of plants and pollinating insects (there is never a scientific name or reference). How can one discuss a network without taking into account the entities involved? In addition, although the authors should be commended for the amount of analysis they have done, there are some gaps in the presentation and clarity. I would recommend summarising the information and accompanying it with a few informative graphs and tables. The conclusions are very vague; for example, there are no suggestions for urban green management, no examples of “virtuous plants” or “rejected plants” in these networks. In short, the taxonomic and applicative part is practically absent.

I would suggest the authors to include more recent and relevant literature about their topic, especially given the fact that they have included barely any references to other urban plant-pollinator networks in Europe. https://doi.org/10.1111/1365-2435.13642, https://doi.org/10.1016/j.agee.2021.107560, https://doi.org/10.1556/168.2017.18.1.1, https://doi.org/10.1111/1365-2664.14168, https://doi.org/10.1016/j.ufug.2024.128650

There are also other more specific comments, which can be found in the bulleted list below. The authors are free to accept or reject my comments following adequate explanation or justification.

Title and Abstract: The title does not convey the main findings of the study. The abstract is ok.

Introduction: The introduction provides with enough detail to understand the topic of the study. I have only some minor comments.

• When pointing out the hypotheses, I would not separate them from the question, but I would rather continue on the same line. In addition, the questions may also be completely removed to keep only the working hypotheses.

• A key point: in the introduction is not mentioned the focal insect taxa of the study, or all insects? Or all animals?

Methods: The sampling method part needs to be clarified because all the information are randomly placed in the text (or missing) in my opinion. Clearly separate a section for the plant surveys, and a section for pollinator counting. How were the pollinators identified and counted? At which taxonomic resolution? How the authors dealt with the possibility of pseudo-replication in pollinators?

• 20-m wide or long? Transects are usually 2m wide and 50-100m long

• “for each plant” … species I would guess no?

• How can the authors avoid double counting if they just visually counted the pollinators? How can you distinguish between two individuals of the same species from the same individual? All this part is very confusing to me.

• “We sampled 62 plant taxa” … this is a result, not a pre-requisite (method) of the study.

• If those two parks were rather poor in plant taxa, why just not excluding them? The number of parks would still be high enough for robust analysis.

• Chao1 index: How were the pollinators identified and counted? At which taxonomic resolution? How the authors dealt with the possibility of pseudo-replication in pollinators? Presence (0) – absence (1). Because how can you record abundance if you just visually monitored the insects. Again, are you dealing with insects or all animals?

• Appendix S2: No, I would suggest putting the definition of the metrics here in the main text, they are essential to the readers to understand the study.

• In building GLMMs, have the authors checked for collinearity? Because I would think that in May-August (period) one should also have higher flower abundance; but these two predictors have been included seemingly blindly.

• I understand the usage of AIC, but this comes with a non-negligible problem: different metrics on the same network are analysed in different ways, which can lead to misleading interpretations. Why do not choose a single model that answers all the questions posed by the authors and use it straightforward? This would also simplify the reading and understanding of the results.

Results: The presentation of the results is insufficient. There are no tables... and the text does not even contain the minimum parameters needed to understand the statistical comparisons/models. Everything needs to be formalised. In this form, the results are barely understandable. The more if you consider that the authors have done a lot of analysis, but in this form, everything is very confusing. I suggest the authors reducing and formalising this section, making it more precise. Now there are a lot of statements, supported by supplementary tables that are not immediately readable. The results should be concise, accompanied by a few extremely significant and informative tables and figures and, given that the authors have carried out numerous analyses, divided by “hypothesis” or “question”.

• Fig.S2 is almost incomprehensible. First, the four periods are in square 2x2 (which I figured out after a while), second there are no names about the interacting species. At the moment, this figure does not provide relevant information and can therefore be removed or refined.

• Fig.1 please add statistically significant comparisons to the boxplots for all the comparisons OR delete plots showing non-significant comparisons. In addition, where are the tables with the selected models? They were not even mentioned in the text the summary statistics of the models

• Table S4. This table should be placed in the text. Why there is never Period 3? In addition, I think here there is a problem of interpretation. When interactions are statistically significant, they should be discussed as the sole significant terms, and not together with their non-interacting factors. You must interpret the interaction first. The main effects become less relevant or misleading because the effect of that variable isn't consistent (as suggested by the significant interaction).

• Fig.3 is almost incomprehensible because it does not convey clear information but rather a chaotic representation of the networks though the four seasons. In addition, again, without names it is even less informative.

Discussion: I believe that, in light of what I have read, the entire discussion and study have a major flaw. The authors did not consider the type of plant (the species are never mentioned) or its floral morphology, nor did they consider the type of insect that visited it. How can one generalise without knowing what types of insects visit what types of flowers? Again, the discussion should be more concise and point only to the hypothesis stated. In addition, no mention about urban green management was done.

• “native and non-native plants differ in several” … It is too vague, state which indices differ

• “the number of species and interactions that persist across several periods” … but this is unlikely since pollinator species that persist throughout the year or flowering period are few and often not “strong” pollinators. Solitary wild bees have flight periods of around 2-3 months at most. Only eusocial species cover the entire season with their workers. This applies only if the analysis were done with pollinators identified at species-level, but I don’t think so.

• “Urban parks represent artificial assemblages of ornamental plants” … but this is not true, or at least not always true. There are a lot of native plants in urban parks as well.

Reviewer #2: In this study, the authors investigate the dynamics of non-native plant integration into plant–pollinator interaction networks in urban parks of a Mediterranean city over the course of one year. They compare native and non-native plants in terms of their structural roles within the network and their contribution to temporal connectivity and interaction turnover. The authors show that native and non-native plants play distinct structural roles while contributing similarly to the temporal connectivity of the network.

At a time when biological invasions are becoming increasingly frequent and may alter interactions between native plants and pollinators, this study is highly relevant. Overall, I found the manuscript to be well structured, timely, and interesting. However, I have a few minor concerns that I would like to share with the authors to help improve the clarity and robustness of the manuscript. Finally, I congratulate the authors on their effort and hope that my comments will be useful.

Minnor comments

Methods

Parks were used as a random factor in the analyses, but it is not clear how this variable varies. Could you better explain this in the Methods section?

Were ruderal/spontaneous plants sampled? Please clarify this point and provide a clear definition of what was considered an ornamental plant in the study.

At which taxonomic level were plants and pollinators identified (e.g., species, genus)? Please clarify this information.

Did you calculate sampling effort for each park? I suggest estimating and reporting this information, possibly as a table in the Supplementary Material.

Did you calculate the sampling completeness of the interaction networks? Although network size was included as a random factor, it would be informative to show how well sampled these networks were.

Why did you use a binary interaction matrix to calculate centrality metrics? Given that you appear to have interaction frequency data, it would be interesting to use quantitative matrices and explore weighted metrics (e.g., weighted closeness and weighted betweenness). The same point applies to beta diversity analyses. Why were quantitative interaction matrices not used to calculate interaction’s beta diversity?

Results

Please indicate the total number of pollinator species recorded in the study. In addition, a brief description comparing plants, pollinators, and interaction diversity among the parks would help to better contextualize the results.

Discussion

The finding that non-native plants tend to overlap less in flowering with other non-native plants is very interesting and could be highlighted more explicitly in the Discussion.

Please provide more details on how the relationships among native–native, non-native–non-native, and native–non-native plant interactions relate to competition and facilitation processes.

Supplementary Material

The title of Table S1 should be placed above the table, not below it.

For the model including within-degree z, the AIC of the model with flowering length as the only predictor and park as a random factor is lower than the others (AIC = 540.8312). If this interpretation is correct, this should be considered the best model, and the corresponding results (Table S3, Table S4, and related text) may need to be adjusted. Why did you not use the delta AIC?

Figures

Please standardize terminology across all figures (e.g., “Non-native” vs. “Nonnative”).

In addition, the different levels of categorical variables should start with capital letters (e.g., “Mix”, “Native”, “Non-native”).

Data availability

I strongly suggest that the authors provide a list of plant species, pollinator species, and recorded interactions. I was not able to access the Zenodo link provided, so I could not verify whether these data are already available.

**********

6. PLOS authors have the option to publish the peer review history of their article (what does this mean?). If published, this will include your full peer review and any attached files.

If you choose “no”, your identity will remain anonymous but your review may still be made public.

**Do you want your identity to be public for this peer review?**  For information about this choice, including consent withdrawal, please see our Privacy Policy.

Reviewer #1: No

Reviewer #2: No

**********

[NOTE: If reviewer comments were submitted as an attachment file, they will be attached to this email and accessible via the submission site. Please log into your account, locate the manuscript record, and check for the action link "View Attachments". If this link does not appear, there are no attachment files.]

To ensure your figures meet our technical requirements, please review our figure guidelines: https://journals.plos.org/plosone/s/figures

You may also use PLOS’s free figure tool, NAAS, to help you prepare publication quality figures: https://journals.plos.org/plosone/s/figures#loc-tools-for-figure-preparation.

NAAS will assess whether your figures meet our technical requirements by comparing each figure against our figure specifications.

Attachment Submitted filename: Comments_PONE-D-25-67039.pdf

# Author comment: Author response to Decision Letter 1

**DOI:** 10.1371/journal.pone.0353207.r002

17 Mar 2026

Dear Prof. Vicente Martínez López,

We are pleased to submit the revised version of our manuscript (PONE-D-25-67039) now entitled “Non-native plant integration into plant-insect pollinator networks in urban parks” for your consideration as a Research Article in PLOS ONE.

We thank the associate editor and the two referees for their general positive and insightful comments. We have revised the manuscript in light of these comments, and we believe these changes have strengthened the new version of the manuscript.

Some of the requests made are presented in the recently published paper titled “Year-round pollinator visitation of ornamental plants in Mediterranean urban parks” by Trillo et al. 2026. Trillo et al. 2026 and our manuscript use the same dataset but do not overlap in their aims and content. Trillo et al. 2026 focus on the identity of the plants and pollinators and management practices in urban green areas, while our focus is on the structure of plant-pollinator networks. Therefore, some of the questions made by referees related to the natural history of the species and sampling methods have not been extensively described in our manuscript but referred to Trillo et al. 2026.

We provide below responses to the suggestions raised by the associate editor and reviewers’ comments, with a full accounting of the changes.

Thank you for your time and consideration.

On behalf of all co-authors, yours sincerely,

Laura Matas-Granados

Dear Dr. Matas-Granados,

Thank you for submitting your manuscript to PLOS ONE. After careful consideration, we feel that it has merit but does not fully meet PLOS ONE’s publication criteria as it currently stands. Therefore, we invite you to submit a revised version of the manuscript that addresses the points raised during the review process.

Both reviewers acknowledge the interest and relevance of the topic addressed in your manuscript. However, several issues require clarification before the manuscript can be further considered. In the revised version, please include line numbers to facilitate the evaluation process. In particular, some methodological aspects need to be described more clearly. The assumption of independence among pollinator samplings is mentioned but should be explained more explicitly. Additionally, please specify the focal insect group considered in the study (e.g., all pollinators, bees only or bees and hoverflies). Overall, I believe that after a careful revision addressing these points, the manuscript could become suitable for publication.

[Response]: We are grateful for your positive assessment of our work. We are very sorry for the lack of line numbers in the previous version of the manuscript. In this new version, we have included them. We provide below responses to the reviewers’ comments to clarify the sampling design and other methodological details. We also indicate the focal insect groups of study (Hymenoptera, Diptera, Lepidoptera and Coleoptera).

Reviewer #1: Comments to the MS: PONE-D-25-67039

I was given the manuscript entitled “Dynamics of non-native plant integration into plant-pollinator networks in urban parks” The manuscript deals with the role on native and non-native flowering plants in pollination network in Sevilla (Spain). I would point out that without line numbers, the process of reviewing gets incredibly more difficult as I have no means of indicating the point of my comments. I would kindly ask the authors to insert line numbers in the other rounds of revision, thanks.

[Response]: We are very sorry about the omission of line numbers. In this new version, we have inserted them.

General opinion: This study aims to understand the temporal dynamics between plants and pollinators in a city over the course of a year, which is a nice new element supporting the study. However, I believe that it cannot be published in its current form because it is too vague in its identification of plants and pollinating insects (there is never a scientific name or reference). How can one discuss a network without taking into account the entities involved? In addition, although the authors should be commended for the amount of analysis they have done, there are some gaps in the presentation and clarity. I would recommend summarising the information and accompanying it with a few informative graphs and tables. The conclusions are very vague; for example, there are no suggestions for urban green management, no examples of “virtuous plants” or “rejected plants” in these networks. In short, the taxonomic and applicative part is practically absent.

I would suggest the authors to include more recent and relevant literature about their topic, especially given the fact that they have included barely any references to other urban plant-pollinator networks in Europe. https://doi.org/10.1111/1365-2435.13642, https://doi.org/10.1016/j.agee.2021.107560, https://doi.org/10.1556/168.2017.18.1.1, https://doi.org/10.1111/1365-2664.14168, https://doi.org/10.1016/j.ufug.2024.128650

[Response]: We thank the reviewer for these constructive comments on our work.

We need to clarify that the main objective of our work was to explore the differences between non-native and native plants in structuring plant-pollinator networks within an experimental system (i.e. urban green areas) that could simulate a potential highly-invaded natural ecosystem. Our intention was not to give descriptive details of species identities nor to develop recommendations for urban management, but rather to compare the role of plant origin on the architecture of plant-pollinator networks and to discuss their potential implications in the context of biological invasion. Our focus is thus on changes in variables at the community level rather than at the species level. Most of the studies suggested by the reviewer focused on the effects of urbanization gradients, urban habitat characteristics, or management practices without considering plant origin, which is our main aim. We have referred studies considering plant origin in urban green areas (e.g. Zaninotto et al. 2023).

The recently published paper Trillo et al. 2026 use the same dataset to focus directly on species composition and urban management practices in these Mediterranean urban green areas. This paper and our work do not overlap in main aims and content. Trillo et al. 2026 focus on the natural history and phenology of species, while our work focusses on the structure of the networks. Nevertheless, we have revised the manuscript to give more detailed information on species taxa (new Figure S2 with species names), and refer to Trillo et al. 2026 whenever required.

There are also other more specific comments, which can be found in the bulleted list below. The authors are free to accept or reject my comments following adequate explanation or justification.

[Response]: Thank you very much. We have included our point-by-point responses hereafter.

Title and Abstract: The title does not convey the main findings of the study. The abstract is ok.

[Response]: We have simplified the title to “Non-native plant integration into plant-insect pollinator networks in urban parks” (lines 1-2),

Introduction: The introduction provides with enough detail to understand the topic of the study. I have only some minor comments.

• When pointing out the hypotheses, I would not separate them from the question, but I would rather continue on the same line. In addition, the questions may also be completely removed to keep only the working hypotheses.

[Response]: We have decided to keep questions to help structuring the headings in methods and results. However, we placed hypothesis on the same line than the question (lines 111-131).

• A key point: in the introduction is not mentioned the focal insect taxa of the study, or all insects? Or all animals?

[Response]: We have also included in the introduction the focal insect taxa (lines 107-109):

“We focus on Hymenoptera, Diptera, Lepidoptera and Coleoptera, which are the main pollinators visiting flowers in Mediterranean urban green areas (Silva et al. 2023).”

Methods: The sampling method part needs to be clarified because all the information are randomly placed in the text (or missing) in my opinion. Clearly separate a section for the plant surveys, and a section for pollinator counting. How were the pollinators identified and counted? At which taxonomic resolution? How the authors dealt with the possibility of pseudo-replication in pollinators?

[Response]: A full description of the sampling method is now published in Trillo et al. 2026. We respond to your questions below. However, in this new version we did not explain the sampling protocol in detail but summarize it as follows (lines 134-149):

“The study was conducted between October 2021 and September 2022 in 15 urban green areas (i.e., parks) within the city of Seville, in southern Spain. In this city, owing to gardening and consistently warm temperatures throughout the year, floral resources are available nearly year-round. In each park, flowering ornamental woody plant species (both native and non-native) were sampled once a month. The plant and insect pollinator sampling protocol are described in Trillo et al. 2026. In sum, in each park flowering plant species which attracted pollinators were sampled across the entire area of the park. Each month, for each plant species, pollinators were sampled in two censuses located in two randomly selected plants along the park. In a census, a 10-minute pollinator observation over a 1 m2 area of the flowering plant species was conducted. Within a census any Hymenoptera, Diptera (>3 mm size), Lepidoptera and Coleoptera (>3 mm size) that contacted any reproductive part of a flower was identified and counted, avoiding double counting. Most specimens were identified visually in the field to the lowest possible taxonomic level, except 7.5% of them that were captured for identification in the laboratory with the help of an expert (Francisco P. Molina). Total sampling was 258 hours across 64 days (S1 Table).”

• 20-m wide or long? Transects are usually 2m wide and 50-100m long

[Response]: We sampled plants along a representative 20 m-wide path, which covered the entire area of the park. We have rewritten this part of the methods and refer to Trillo et al. 2026 when needed.

• “for each plant” … species I would guess no?

[Response]: From each species, we sampled pollinators on several plants. It is thus correct to say that during each census, we conducted a 10-min pollinator count per plant.

• How can the authors avoid double counting if they just visually counted the pollinators? How can you distinguish between two individuals of the same species from the same individual? All this part is very confusing to me.

[Response]: As in most studies based on visual observations of flower visitors, completely avoiding the possibility of recounting the same individual is almost impossible. However, during the surveys, we attempted to minimize double counting by tracking individual movements within the same focal plant. When a pollinator of a given species was observed moving between nearby flowers and returning to the same plant, it was not counted again. It is possible that in some cases the same individual may have been recorded more than once in different focal plants or species. However, each observation period lasted only 10 minutes and multiple flowering plants were available in the surroundings, which reduces the likelihood that the same individual repeatedly visited the same focal plant within the sampling period. Further, pollinators usually avoid recently visited flowers (Jones et al. 1998). In any case, any potential overestimation of visitation rates would affect all plants and parks in a similar way, meaning that relative comparisons among parks remain robust.

• “We sampled 62 plant taxa” … this is a result, not a pre-requisite (method) of the study.

[Response]: We placed the number of taxa in methods because now we refer to the sampling and identification of species described in Trillo et al. 2026.

• If those two parks were rather poor in plant taxa, why just not excluding them? The number of parks would still be high enough for robust analysis.

[Response]: These two parks did not receive pollinator visits during two of the four periods considered. However, we chose to retain them in the analyses in order to preserve the full range of variation in plant-pollinator interactions and to avoid selectively excluding parks with low pollination activity in some months of the year. Having said that, if the reviewer and the editor consider their exclusion necessary to ensure the robustness of our results, we are open to repeat the analyses without these two parks and report the results accordingly.

• Chao1 index: How were the pollinators identified and counted? At which taxonomic resolution? How the authors dealt with the possibility of pseudo-replication in pollinators? Presence (0) – absence (1). Because how can you record abundance if you just visually monitored the insects. Again, are you dealing with insects or all animals?

[Response]: The pollinators were identified at the species level by visual observation in the field by Trillo et al. 2026 or in the laboratory by an expert (Francisco P. Molina) when identification was not possible in the field.

Abundance was estimated in a 1 m2 area with the highest flower density for 10 minutes. See our response above related to avoiding double counting in pollinators.

• Appendix S2: No, I would suggest putting the definition of the metrics here in the main text, they are essential to the readers to understand the study.

[Response]: In this new version, we have included the description of the species-level network parameters in the main text (lines 173-196).

• In building GLMMs, have the authors checked for collinearity? Because I would think that in May-August (period) one should also have higher flower abundance; but these two predictors have been included seemingly blindly.

[Response]: We agree about testing the potential collinearity between predictors. We assessed collinearity among predictors using generalized variance inflation factors (GVIFs) for models with some categorical predictors (Fox & Monette 1992). All values for all models were below 1.6, indicating no problematic collinearity among predictors. Therefore, including period and floral availability in the models does not affect the interpretation of the best model. We included this aspect in the methodology (lines 223-225):

“We assessed potential collinearity among predictors for models using generalized variance inflation factors (GVIFs) (Fox & Monette 1992). All values for all models were below 1.6, indicating no problematic collinearity among predictors.”

• I understand the usage of AIC, but this comes with a non-negligible problem: different metrics on the same network are analysed in different ways, which can lead to misleading interpretations. Why do not choose a single model that answers all the questions posed by the authors and use it straightforward? This would also simplify the reading and understanding of the results.

[Response]: Thank you for this consideration. Selecting models using AIC for each species-level network parameter is the most statistically robust approach, since each species-level network parameter explain a different characteristic of the network structure. Therefore, each parameter may have related to different predictors, and forcing a single model for all metrics could lead to overfitting or the inclusion of irrelevant predictor for some parameters. Nevertheless, we understand the complexity in the results considering a different best model for each parameter and we have made an effort reducing non-significant results in new Figure 1.

Results: The presentation of the results is insufficient. There are no tables... and the text does not even contain the minimum parameters needed to understand the statistical comparisons/models. Everything needs to be formalised. In this form, the results are barely understandable. The more if you consider that the authors have done a lot of analysis, but in this form, everything is very confusing. I suggest the authors reducing and formalising this section, making it more prec

Attachment Submitted filename: Response to Reviewers.docx

# Peer review: Decision Letter 1

**DOI:** 10.1371/journal.pone.0353207.r003

**Authors:** Vicente Martínez López, Vicente Martínez López

11 May 2026

PONE-D-25-67039R1

Non-native plant integration into plant-insect pollinator networks in urban parks

PLOS One

Dear Dr. Matas-Granados,

Thank you for submitting your manuscript to PLOS ONE. After careful consideration, we feel that it has merit but does not fully meet PLOS ONE’s publication criteria as it currently stands. Therefore, we invite you to submit a revised version of the manuscript that addresses the points raised during the review process.

Please submit your revised manuscript by Jun 25 2026 11:59PM. If you will need more time than this to complete your revisions, please reply to this message or contact the journal office at plosone@plos.org. When you're ready to submit your revision, log on to https://www.editorialmanager.com/pone/ and select the 'Submissions Needing Revision' folder to locate your manuscript file.

Please include the following items when submitting your revised manuscript:

- A letter that responds to each point raised by the academic editor and reviewer(s). You should upload this letter as a separate file labeled 'Response to Reviewers'.
- A marked-up copy of your manuscript that highlights changes made to the original version. You should upload this as a separate file labeled 'Revised Manuscript with Track Changes'.
- An unmarked version of your revised paper without tracked changes. You should upload this as a separate file labeled 'Manuscript'.

If you would like to make changes to your financial disclosure, please include your updated statement in your cover letter. Guidelines for resubmitting your figure files are available below the reviewer comments at the end of this letter.

If applicable, we recommend that you deposit your laboratory protocols in protocols.io to enhance the reproducibility of your results. Protocols.io assigns your protocol its own identifier (DOI) so that it can be cited independently in the future. For instructions see: https://journals.plos.org/plosone/s/submission-guidelines#loc-laboratory-protocols. Additionally, PLOS ONE offers an option for publishing peer-reviewed Lab Protocol articles, which describe protocols hosted on protocols.io. Read more information on sharing protocols at https://plos.org/protocols?utm_medium=editorial-email&utm_source=authorletters&utm_campaign=protocols.

As the corresponding author, your ORCID iD is verified in the submission system and will appear in the published article. PLOS supports the use of ORCID, and we encourage all coauthors to register for an ORCID iD and use it as well. Please encourage your coauthors to verify their ORCID iD within the submission system before final acceptance, as unverified ORCID iDs will not appear in the published article. *Only*  the individual author can complete the verification step; PLOS staff *cannot*  verify ORCID iDs on behalf of authors.

We look forward to receiving your revised manuscript.

Kind regards,

Vicente Martínez López

Academic Editor

PLOS One

Journal Requirements:

1. If the reviewer comments include a recommendation to cite specific previously published works, please review and evaluate these publications to determine whether they are relevant and should be cited. There is no requirement to cite these works unless the editor has indicated otherwise.

2. Please review your reference list to ensure that it is complete and correct. If you have cited papers that have been retracted, please include the rationale for doing so in the manuscript text, or remove these references and replace them with relevant current references. Any changes to the reference list should be mentioned in the rebuttal letter that accompanies your revised manuscript. If you need to cite a retracted article, indicate the article’s retracted status in the References list and also include a citation and full reference for the retraction notice.

Additional Editor Comments:

Both reviewers acknowledge the improvement of the manuscript after revision. However, they still identify several issues that should be addressed before the manuscript can be considered for publication. In particular, Reviewer 1 suggests further improvements to the Discussion section, while Reviewer 2 highlights some aspects of the methodology and results that require clarification. Please carefully address these remaining concerns in a revised version of the manuscript, and I will be pleased to reconsider it for publication in PLOS ONE.

[Note: HTML markup is below. Please do not edit.]

Reviewer's Responses to Questions

**Comments to the Author**

1. If the authors have adequately addressed your comments raised in a previous round of review and you feel that this manuscript is now acceptable for publication, you may indicate that here to bypass the “Comments to the Author” section, enter your conflict of interest statement in the “Confidential to Editor” section, and submit your "Accept" recommendation.

Reviewer #1: All comments have been addressed

Reviewer #2: All comments have been addressed

**********

2. Is the manuscript technically sound, and do the data support the conclusions?

The manuscript must describe a technically sound piece of scientific research with data that supports the conclusions. Experiments must have been conducted rigorously, with appropriate controls, replication, and sample sizes. The conclusions must be drawn appropriately based on the data presented.

Reviewer #1: Yes

Reviewer #2: Yes

**********

3. Has the statistical analysis been performed appropriately and rigorously?

Reviewer #1: Yes

Reviewer #2: Yes

**********

4. Have the authors made all data underlying the findings in their manuscript fully available?

The PLOS Data policy requires authors to make all data underlying the findings described in their manuscript fully available without restriction, with rare exception (please refer to the Data Availability Statement in the manuscript PDF file). The data should be provided as part of the manuscript or its supporting information, or deposited to a public repository. For example, in addition to summary statistics, the data points behind means, medians and variance measures should be available. If there are restrictions on publicly sharing data—e.g. participant privacy or use of data from a third party—those must be specified.

Reviewer #1: Yes

Reviewer #2: Yes

**********

5. Is the manuscript presented in an intelligible fashion and written in standard English?

PLOS ONE does not copyedit accepted manuscripts, so the language in submitted articles must be clear, correct, and unambiguous. Any typographical or grammatical errors should be corrected at revision, so please note any specific errors here.

Reviewer #1: Yes

Reviewer #2: Yes

**********

6. Review Comments to the Author

Please use the space provided to explain your answers to the questions above. You may also include additional comments for the author, including concerns about dual publication, research ethics, or publication ethics. (Please upload your review as an attachment if it exceeds 20,000 characters)

Reviewer #1: Comments to the MS: PONE-D-25-67039_R1

I was given the revised version of the manuscript now entitled “Non-native plant integration into plant-insect pollinator networks in urban parks”

Overall, the manuscript has been improved and I think, if other reviewers and editors agree, merits publication. I have only few minor comments that the author can easily resolve without a rebuttal letter.

The introduction was already well presented and therefore didn’t need any further comments.

The methods are now cleared and better divided into subsections. While I do not like referencing to other papers in the methods (because I would like to read it in the paper I selected), I understand the methods are those from Trillo. I don’t like the choice, but I respect it.

• L212: Km 2

The results are now clearer with these subheadings.

• In table 1, Among-module connectivity, P = 0.055 should not be boldfaced, it is not statistically significant.

The discussion should be adjusted. First, I would not separate the discussion into subsections, but I would rather make a cohesive, concise (!!!), and informative section. I recommend the authors to short the discussion to really highlight what they have found. Instead use paragraphs to separate arguments. Furthermore, I must unfortunately accept that I will not see any plant names listed, nor any mention of their potential positive or negative role within the network. I find it a major shortcoming that no plants are identified as keystone species for urban networks. This study fails to offer any practical applications, despite its great potential to do so. Instead, however, it engages in extensive speculation on purely theoretical network concepts, which I find of little interest.

• L385: six rows for a single sentence are way too much, please rephrase.

Reviewer #2: The authors have done an excellent job in improving this new version of the manuscript. The study is now much more consistent with the questions and hypotheses being tested, and I would like to congratulate them on these improvements.

However, I still have a few concerns. As the authors note, this study and Trillo et al. (2026) used the same dataset. Although the main objective of the present work is not directly focused on species identity or management practices in urban areas, these aspects are highly relevant when working with interaction networks in urban green species. In this context, the inclusion of species names in the bipartite networks is an important and welcome improvement.

In addition, I strongly suggest the inclusion of two new supplementary tables: one presenting the plant species along with relevant traits (e.g., origin), and another with information on the pollinators. I also encourage the authors to make the interaction data publicly available (e.g., via Zenodo).

The study relies on visual observations, and as nicely pointed out by Reviewer 1, it is still unclear how the models address the issue of pseudoreplication. If this issue is not accounted for in the analyses, I suggest that it be explicitly acknowledged as a limitation of the study.

Regarding the methods, it would be helpful to clarify whether the sampling design was based on a transect or a plot. If it was a transect, as it seems, please specify its length, given that its width is reported as 20 m.

Line 337: Please check the results, as the nested contribution does not appear to decrease with network size.

Table S1: I suggest including the standard deviation in the column “Mean time census per plant species” to better represent the variation.

Figure S2: Please clarify in the legend what the colours in the boxes represent for each species, especially for plants. It would be helpful to indicate whether the colours correspond to species origin (native vs. non-native), as this seems to be the case but is not explicitly stated. For pollinators, you may consider colouring the boxes according to the different orders recorded (e.g., Hymenoptera, Diptera, Lepidoptera, and Coleoptera).

**********

7. PLOS authors have the option to publish the peer review history of their article (what does this mean?). If published, this will include your full peer review and any attached files.

If you choose “no”, your identity will remain anonymous but your review may still be made public.

**Do you want your identity to be public for this peer review?**  For information about this choice, including consent withdrawal, please see our Privacy Policy.

Reviewer #1: No

Reviewer #2: No

**********

[NOTE: If reviewer comments were submitted as an attachment file, they will be attached to this email and accessible via the submission site. Please log into your account, locate the manuscript record, and check for the action link "View Attachments". If this link does not appear, there are no attachment files.]

To ensure your figures meet our technical requirements, please review our figure guidelines: https://journals.plos.org/plosone/s/figures

You may also use PLOS’s free figure tool, NAAS, to help you prepare publication quality figures: https://journals.plos.org/plosone/s/figures#loc-tools-for-figure-preparation.

NAAS will assess whether your figures meet our technical requirements by comparing each figure against our figure specifications.

Attachment Submitted filename: Comments_PONE-D-25-67039_R1.docx

# Author comment: Author response to Decision Letter 2

**DOI:** 10.1371/journal.pone.0353207.r004

22 May 2026

Dear Prof. Vicente Martínez López,

Thank you for your assessment and positive feedback from both reviewers regarding the improvements made in the revised version.

We have carefully considered all remaining comments and concerns raised by the reviewers. In response, we have further strengthened the Discussion section, as suggested by Reviewer 1, clarified the methodology and added new tables as recommended by Reviewer 2. Detailed responses to each comment, together with the corresponding changes made in the manuscript, are provided in the revised response document.

We hope that the revised version will now be suitable for publication in Plos ONE.

Laura Matas-Granados

Dear Dr. Matas-Granados,

Both reviewers acknowledge the improvement of the manuscript after revision. However, they still identify several issues that should be addressed before the manuscript can be considered for publication. In particular, Reviewer 1 suggests further improvements to the Discussion section, while Reviewer 2 highlights some aspects of the methodology and results that require clarification. Please carefully address these remaining concerns in a revised version of the manuscript, and I will be pleased to reconsider it for publication in PLOS ONE.

[Response]: Thank you for the assessment and the positive feedback. Find below our responses to reviewers’ comments.

Reviewer #1: Comments to the MS: PONE-D-25-67039_R1

I was given the revised version of the manuscript now entitled “Non-native plant integration into plant-insect pollinator networks in urban parks”

Overall, the manuscript has been improved and I think, if other reviewers and editors agree, merits publication. I have only few minor comments that the author can easily resolve without a rebuttal letter.

The introduction was already well presented and therefore didn’t need any further comments.

The methods are now cleared and better divided into subsections. While I do not like referencing to other papers in the methods (because I would like to read it in the paper I selected), I understand the methods are those from Trillo. I don’t like the choice, but I respect it.

[Response]: Thank you for the assessment. To improve clarity and make the methodology easier to follow for readers; we have now transcribed verbatim the sampling design methodology originally described by Trillo et al. 2026 into the Supplementary Material (S2 Appendix). We hope that this addition will not be considered plagiarism. We are open to remove it if you consider it.

We added in the text:

Lines 152-154: “For further detailed information regarding the sampling design, please refer to Trillo et al. 2026 (transcribed verbatim in S2 Appendix)”.

• L212: Km 2

[Response]: We have changed it accordingly.

The results are now clearer with these subheadings.

• In table 1, Among-module connectivity, P = 0.055 should not be boldfaced, it is not statistically significant.

[Response]: We have not boldfaced marginal significant terms; instead, we indicated them with a “+” symbol.

The discussion should be adjusted. First, I would not separate the discussion into subsections, but I would rather make a cohesive, concise (!!!), and informative section. I recommend the authors to short the discussion to really highlight what they have found. Instead use paragraphs to separate arguments. Furthermore, I must unfortunately accept that I will not see any plant names listed, nor any mention of their potential positive or negative role within the network. I find it a major shortcoming that no plants are identified as keystone species for urban networks. This study fails to offer any practical applications, despite its great potential to do so. Instead, however, it engages in extensive speculation on purely theoretical network concepts, which I find of little interest.

[Response]: Following your recommendation, we have revised and shortened the Discussion by 27% to improve its clarity and conciseness. We removed the previous subsection structure and reduced extensive theoretical considerations on network concepts in order to focus more directly on the main findings of the study.

In addition, we incorporated specific examples of plant species and key plant–pollinator interactions identified in our results to better support our interpretations and increase the practical relevance of the study:

Lines 399-401: “However, contrary to our hypothesis, non-native plants, such as Pyracantha coccinea or Lantana spp., displayed greater specialization than native plants during resource-scarce periods.”

Lines 404-407: “In contrast, native plants exhibited greater species strength (e.g., Salvia rosmarinus), indicating that a wide range of pollinators depend on them when available, highlighting their role in sustaining network stability.”

Lines 408-410: “As expected, non-native plants showed higher among-module connectivity (e.g., Agapanthus africanus), whereas natives exhibited higher within-module degree (e.g., Salvia rosmarinus).”

Lines 420-423: “However, native–non-native pairs forming modules tended to co-flower more frequently than non-native–non-native pairs. This is illustrated by examples such as the pair Lavandula spp. (native) and Lantana spp. (non-native) which co-flowered during three of the four periods.”

Lines 450-454: “This high turnover aligns with the finding that only a few interactions connected temporal networks over the course of the year. This will be the case for some interactions between Apis mellifera and Xylocopa violacea with both native (e.g., Lavandula spp.) and non-native plants (e.g., Duranta erecta), underscoring the transient nature of most plant–pollinator interactions.

• L385: six rows for a single sentence are way too much, please rephrase.

[Response]: We have simplified it accordingly:

Lines 392-396: “Our study examining year-round plant-pollination interactions in urban green areas indicate that native and non-native plants differ on how they integrate into the plant-pollinator network and in their modules. However, native and non-native plants have similar capacities as temporal network connectors, and on their contribution to interaction turnover.”

Reviewer #2: The authors have done an excellent job in improving this new version of the manuscript. The study is now much more consistent with the questions and hypotheses being tested, and I would like to congratulate them on these improvements.

However, I still have a few concerns. As the authors note, this study and Trillo et al. (2026) used the same dataset. Although the main objective of the present work is not directly focused on species identity or management practices in urban areas, these aspects are highly relevant when working with interaction networks in urban green species. In this context, the inclusion of species names in the bipartite networks is an important and welcome improvement.

In addition, I strongly suggest the inclusion of two new supplementary tables: one presenting the plant species along with relevant traits (e.g., origin), and another with information on the pollinators. I also encourage the authors to make the interaction data publicly available (e.g., via Zenodo).

[Response]: Thank you for the assessment. We have now included in the supplementary information a table with the information of plant species sampled (new S2 Table) and another table with pollinator species sampled (new S3 Table). We have also included as publicly available a table with all the plant-pollinator interactions recorded (“Table_interactions.csv” in https://doi.org/10.20350/digitalCSIC/18370 in DIGITAL CSIC and https://doi.org/10.5281/zenodo.20305055 in Zenodo).

The study relies on visual observations, and as nicely pointed out by Reviewer 1, it is still unclear how the models address the issue of pseudoreplication. If this issue is not accounted for in the analyses, I suggest that it be explicitly acknowledged as a limitation of the study.

[Response]: We avoided counting the same individual visiting different flowers during a census. However, as we mentioned in our previous response, we acknowledge that, as in most studies based on visual observations of flower visitors, it is not possible to completely avoid pseudoreplication, because the same individual pollinator may be recorded more than once if it departs away from the sampling area and comes back during the sampling period. We have now included this limitation of the study.

Lines 144-149: “Within a census any Hymenoptera, Diptera (>3 mm size), Lepidoptera and Coleoptera (>3 mm size) that contacted any reproductive part of a flower was identified and counted. We avoided double counting the same individual within the 1 m2 area sampling area. However, we acknowledge that pseudoreplication could not be entirely excluded if, for instance, the individual leaves the sampling area and comes back.”

Regarding the methods, it would be helpful to clarify whether the sampling design was based on a transect or a plot. If it was a transect, as it seems, please specify its length, given that its width is reported as 20 m.

[Response]: Surveys were conducted in a representative sampling plot of the park. This plot was 20 m-wide with a length that covered the entire extent of each park. Therefore, the length of the plot varied depending on park size, because the objective was to comprehensively sample all flowering woody plant species within each park. We have included in methods:

Lines 137-139: “In each park, we selected a representative 20 m-wide plot with a length that covered the entire extent of each park. In each plot, flowering ornamental woody plant species (both native and non-native) were sampled once a month.”

Line 337: Please check the results, as the nested contribution does not appear to decrease with network size.

[Response]: We have changed it accordingly:

Lines 342-345: “Normalized degree, species strength, and contribution to nestedness increased with flower availability, whereas normalized degree and species strength decreased, and contribution to nestedness increased, with larger network size (Table 1; S4 Fig).”

Table S1: I suggest including the standard deviation in the column “Mean time census per plant species” to better represent the variation.

[Response]: We have added the standard deviation to the mean time census per plant species.

Figure S2: Please clarify in the legend what the colours in the boxes represent for each species, especially for plants. It would be helpful to indicate whether the colours correspond to species origin (native vs. non-native), as this seems to be the case but is not explicitly stated. For pollinators, you may consider colouring the boxes according to the different orders recorded (e.g., Hymenoptera, Diptera, Lepidoptera, and Coleoptera).

[Response]: We have added to the legend the significance of each colours for plant and pollinator taxa. Colours for plant taxa represent their plant origin and now colors for pollinator taxa represent their order.

Attachment Submitted filename: Response_to_Reviewers_auresp_2.docx

# Peer review: Decision Letter 2

**DOI:** 10.1371/journal.pone.0353207.r005

**Authors:** Vicente Martínez López, Vicente Martínez López, Vicente Martínez López

21 Jun 2026

Non-native plant integration into plant-insect pollinator networks in urban parks

PONE-D-25-67039R2

Dear Dr. Matas-Granados,

We’re pleased to inform you that your manuscript has been judged scientifically suitable for publication and will be formally accepted for publication once it meets all outstanding technical requirements.

Within one week, you’ll receive an e-mail detailing the required amendments. When these have been addressed, you’ll receive a formal acceptance letter and your manuscript will be scheduled for publication.

An invoice will be generated when your article is formally accepted. Please note, if your institution has a publishing partnership with PLOS and your article meets the relevant criteria, all or part of your publication costs will be covered. Please make sure your user information is up-to-date by logging into Editorial Manager at Editorial Manager® and clicking the ‘Update My Information' link at the top of the page. For questions related to billing, please contact billing support.

If your institution or institutions have a press office, please notify them about your upcoming paper to help maximize its impact. If they’ll be preparing press materials, please inform our press team as soon as possible -- no later than 48 hours after receiving the formal acceptance. Your manuscript will remain under strict press embargo until 2 pm Eastern Time on the date of publication. For more information, please contact onepress@plos.org.

Kind regards,

Vicente Martínez López

Academic Editor

PLOS One

Additional Editor Comments (optional):

Reviewers' comments:

Reviewer's Responses to Questions

**Comments to the Author**

1. If the authors have adequately addressed your comments raised in a previous round of review and you feel that this manuscript is now acceptable for publication, you may indicate that here to bypass the “Comments to the Author” section, enter your conflict of interest statement in the “Confidential to Editor” section, and submit your "Accept" recommendation.

Reviewer #2: All comments have been addressed

**********

2. Is the manuscript technically sound, and do the data support the conclusions?

The manuscript must describe a technically sound piece of scientific research with data that supports the conclusions. Experiments must have been conducted rigorously, with appropriate controls, replication, and sample sizes. The conclusions must be drawn appropriately based on the data presented.

Reviewer #2: Yes

**********

3. Has the statistical analysis been performed appropriately and rigorously?

Reviewer #2: Yes

**********

4. Have the authors made all data underlying the findings in their manuscript fully available?

The PLOS Data policy requires authors to make all data underlying the findings described in their manuscript fully available without restriction, with rare exception (please refer to the Data Availability Statement in the manuscript PDF file). The data should be provided as part of the manuscript or its supporting information, or deposited to a public repository. For example, in addition to summary statistics, the data points behind means, medians and variance measures should be available. If there are restrictions on publicly sharing data—e.g. participant privacy or use of data from a third party—those must be specified.

Reviewer #2: Yes

**********

5. Is the manuscript presented in an intelligible fashion and written in standard English?

PLOS ONE does not copyedit accepted manuscripts, so the language in submitted articles must be clear, correct, and unambiguous. Any typographical or grammatical errors should be corrected at revision, so please note any specific errors here.

Reviewer #2: Yes

**********

6. Review Comments to the Author

Please use the space provided to explain your answers to the questions above. You may also include additional comments for the author, including concerns about dual publication, research ethics, or publication ethics. (Please upload your review as an attachment if it exceeds 20,000 characters)

Reviewer #2: (No Response)

**********

7. PLOS authors have the option to publish the peer review history of their article (what does this mean?). If published, this will include your full peer review and any attached files.

If you choose “no”, your identity will remain anonymous but your review may still be made public.

**Do you want your identity to be public for this peer review?**  For information about this choice, including consent withdrawal, please see our Privacy Policy.

Reviewer #2: No

**********

# Editor report: Acceptance letter

**DOI:** 10.1371/journal.pone.0353207.r006

**Authors:** Vicente Martínez López, Vicente Martínez López, Vicente Martínez López

PONE-D-25-67039R2

PLOS One

Dear Dr. Matas-Granados,

I'm pleased to inform you that your manuscript has been deemed suitable for publication in PLOS One. Congratulations! Your manuscript is now being handed over to our production team.

At this stage, our production department will prepare your paper for publication. This includes ensuring the following:

* All references, tables, and figures are properly cited

* All relevant supporting information is included in the manuscript submission,

* There are no issues that prevent the paper from being properly typeset

You will receive further instructions from the production team, including instructions on how to review your proof when it is ready. Please keep in mind that we are working through a large volume of accepted articles, so please give us a few days to review your paper and let you know the next and final steps.

Lastly, if your institution or institutions have a press office, please let them know about your upcoming paper now to help maximize its impact. If they'll be preparing press materials, please inform our press team within the next 48 hours. Your manuscript will remain under strict press embargo until 2 pm Eastern Time on the date of publication. For more information, please contact onepress@plos.org.

You will receive an invoice from PLOS for your publication fee after your manuscript has reached the completed accept phase. If you receive an email requesting payment before acceptance or for any other service, this may be a phishing scheme. Learn how to identify phishing emails and protect your accounts at https://explore.plos.org/phishing.

If we can help with anything else, please email us at customercare@plos.org.

Thank you for submitting your work to PLOS ONE and supporting open access.

Kind regards,

PLOS ONE Editorial Office Staff

on behalf of

Dr. Vicente Martínez López

Academic Editor

PLOS One
